# Antigenic mapping of H2 influenza viruses recognized by ferret and human sera and predicting antigenically significant sites

**DOI:** 10.1128/msphere.00022-26

**Published:** 2026-06-10

**Authors:** Z. Beau Reneer, Cameron Ngyen, Matthew R. Corn, Jordan L. Paugh, Owen C. Reynolds, Eric O'Hara, Ted M. Ross, Ralph S. Baric

**Affiliations:** 1 Department of Epidemiology, The University of North Carolina at Chapel Hill 2331, Chapel Hill, North Carolina, USA; 2 Departments of Microbiology and Immunology, The University of North Carolina at Chapel Hill 2331, Chapel Hill, North Carolina, USA; 3 School of Medicine, Mercer University 138565, Savannah, Georgia, USA; 4 Medical College of Georgia 1421 , Augusta, Georgia, USA; 5 Department of Computer Science, University of Georgia 1355, Athens, Georgia, USA; 6 Augusta University/University of Georgia Medical Partnership 427000, Athens, Georgia, USA; 7 Florida Research and Innovation Center, Cleveland Clinic 587918 , Port St. Lucie, Florida, USA; 8 Center for Vaccines and Immunology, University of Georgia 1355, Athens, Georgia, USA; Johns Hopkins University Bloomberg School of Public Health, Baltimore, Maryland, USA

**Keywords:** influenza, H2N2, cartography, viral immunity

## Abstract

Influenza viruses cause hundreds of thousands of infections globally every year. In the past century, seasonal influenza viruses have included H1N1, H2N2, and H3N2 strains. H2N2 influenza viruses circulated in the human population between 1957 and 1968. Previously, our group demonstrated a lack of H2N2 influenza virus immunity in individuals born after 1968, as well as the effectiveness of hemagglutinin (HA)-based vaccines for multiple influenza virus subtypes. In this study, H2 antigenic maps and radial graphs were generated using previously published data from H2 HA vaccinations of ferrets and seasonal influenza vaccinations of humans. The antigenic maps revealed a stark difference in the clustering of HA antigens between ferrets and humans, and the radial graphs showed that specific antigen recognition varies greatly among different influenza preimmune ferrets. These maps also revealed the significant impact that different pre-existing immunities have on antigenic recognition and clustering of antigens after vaccine boost. From these data, we predicted two possible antigenically significant sites containing various mutations that have not been previously reported, and showed that one of these sites is relevant using mouse antisera.

**IMPORTANCE:**

H2N2 influenza viruses have caused at least one known pandemic in humans, and are poised to cause future pandemics. Investigating the antigenic diversity of H2 hemagglutinin (HA) proteins provides valuable data for designing and understanding the performance of current and future vaccines. Data evaluating the differences in antigen recognition across differing pre-existing immunity can be used to predict antigenically significant sites and evaluate the impact of H1 and H3 infection and immune imprinting on H2 vaccine immunogenicity. This information can direct future studies when both extrapolating animal data to human studies and creating next-generation vaccines. Contrasting the relationships among new, contemporary, and ancestral H2 HA antigens by antigenic cartography is imperative for identifying new variants of concern and updating vaccine formulations.

## INTRODUCTION

The 1957 influenza pandemic was caused by a novel H2N2 influenza virus entering the human population (1). This novel H2N2 influenza virus was the result of a reassortment event between an avian H2N2 influenza virus and a human seasonal H1N1 virus strain (2). The new H2N2 influenza virus encoded the PB1, hemagglutinin (HA), and neuraminidase (NA) gene segments from the avian H2N2 influenza virus, while the remaining five gene segments were derived from human H1N1 influenza virus (2, 3). The 1957 pandemic resulted in an estimated 1–2 million deaths worldwide (4). Over the 11 years, H2N2 influenza viruses circulated as seasonal influenza viruses and underwent several antigenic changes in the HA protein before H3N2 replaced H2N2 in the human population in 1968 (5). Thereafter, human H2N2 infections have not been reported despite many avian strains showing efficient replication in primary human bronchial airway cells, foreshadowing the potential for future reemergence events (6, 7).

While H2N2 influenza viruses have not been isolated from humans since 1968, H2Nx influenza viruses have been isolated multiple times from avian species, poultry, swine, and other mammals (7). Additionally, humans are infected with other circulating HA subtypes of influenza viruses (H1N1 and H3N2) throughout their lives (2, 8, 9). Previous exposure(s) to influenza viruses and, in particular, the first influenza virus infection subtype that an individual experiences shape the performance of future influenza virus vaccinations and infections (10–12). Human populations have been infected with different influenza A viruses (H1N1, H2N2, and/or H3N2) over the last 80+ years (2). The antigenic diversity of H2N2 influenza viruses, combined with differences in pre-existing immunity, including the lack of pre-existing immunity in humans under the age of 55, poses enormous challenges for H2N2 vaccine design and testing (13, 14).

Antigenic cartography has provided novel insights into the antigenic relationships among seasonal influenza viruses, SARS-CoV-2, and human noroviruses, all of which evolve rapidly in response to human herd immunity (15–17). In this study, we re-evaluated previously published data to investigate the differences in H2 HA antigen immune recognition (13, 14). Using sera collected from ferrets, with or without pre-existing immunity to influenza A viruses, as well as sera from human subjects (13, 14), antigenic cartography was applied to map antigenic relationships based on different exposure histories, determine the cumulative effect of multiple mutations of the H2 HA proteins, and identify clusters of variants. Additionally, the antigenic and hemagglutination inhibition (HAI) results were re-analyzed to investigate the molecular basis for antigenic drift in human H2N2 viruses. We also evaluated the consequence of the order of infection/sequential infections (e.g., H1N1 and then H3N2 vs H3N2 and then H1N1) on H2 immune breadth and whether immune imprinting after single or multiple HA exposures altered H2 vaccine performance. While H3N2 alone and H3N2+H1N1 restricted H2 breadth after vaccination, we show that both H1N1 alone and H1N1+H3N2 preimmunity promote H2 immune breadth after vaccination. In addition to previously recognized sites in the H2 HA glycoprotein, two additional amino acid residues (sites 140 and 151) were identified that potentially contribute to the HAI and antigenic cartography diversity of the H2 influenza viruses in a strain-specific manner. The significance of these two antigenic sites was determined by generating wild-type (WT) and mutant virus-like particles (VLPs) containing mutations at either site 140 in the H2N2 virus in the strain Muskrat/Russia/14 or site 151 in strain Taiwan/64. Each mutant VLP was probed using antisera from H2 VLP-vaccinated BALB/c mice, demonstrating a 3.5- to 6-fold difference in a residue- and strain-specific manner. These findings provide new insights for designing broadly cross-reactive H2 influenza vaccines.

## RESULTS

### Preimmune ferret antigenic variation of H2 HAs

**Fig 1:**
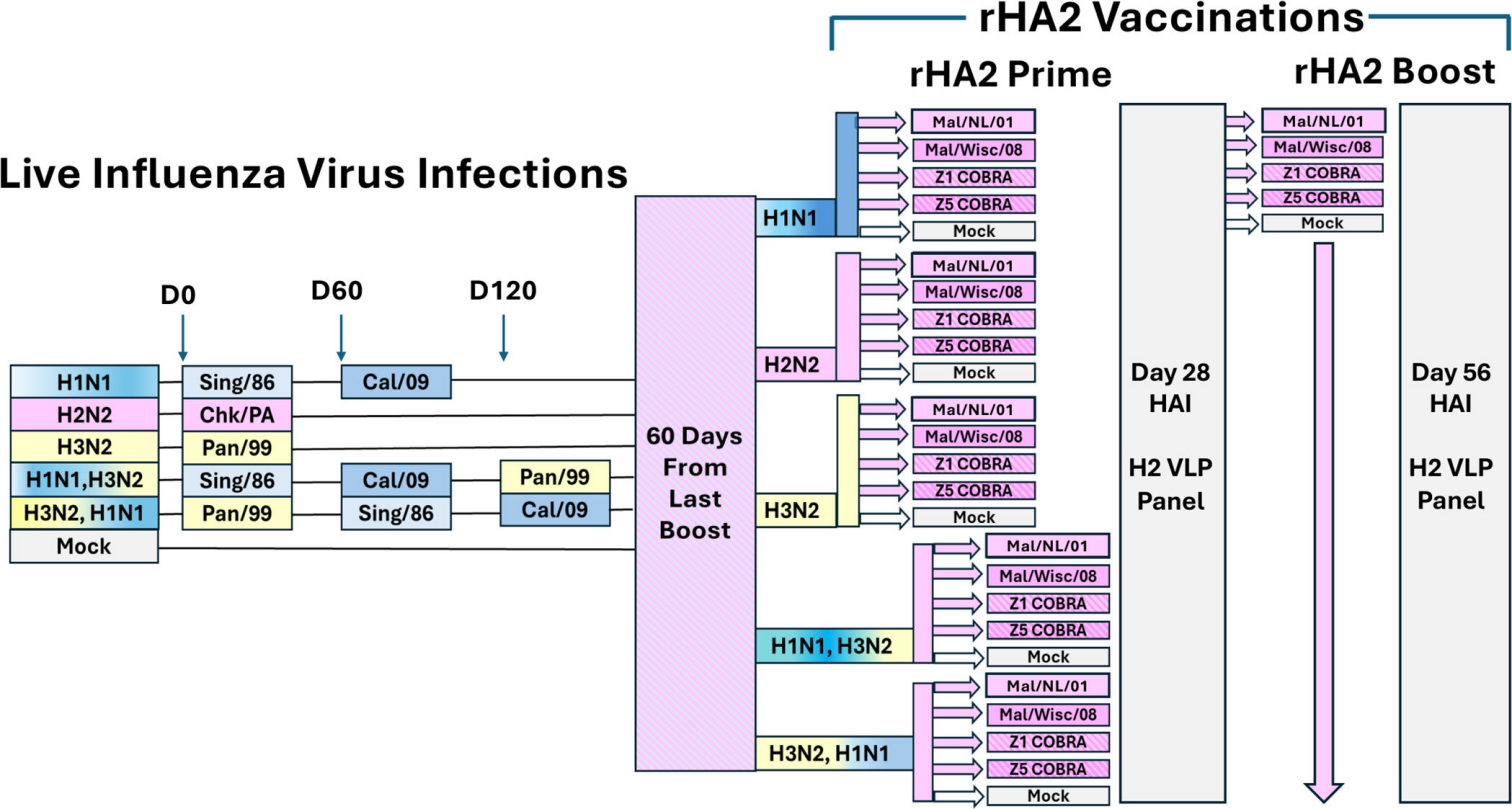
Ferret preimmunity and vaccination outline. Ferrets (*n* = 20/group) were infected with influenza viruses on D0 to establish influenza preimmunity. Ferrets were allowed to rest for 60 days before three groups of ferrets were infected a second time with a heterologous influenza virus. All of the ferrets were allowed to rest for 60 days before two of the three groups of ferrets were infected a third time with a heterologous influenza virus. Sixty days after each ferret group’s final influenza virus infection, ferrets in each preimmune group (*n* = 4) were vaccinated with either one of four rHA vaccines or a mock vaccine containing only PBS and adjuvant. Twenty-eight days after prime vaccination, each ferret was vaccinated with the homologous vaccine that it had received previously.

HAI and neutralization titers in humans and ferrets are commonly used to evaluate the antigenic properties of influenza viruses. Herein, we use cartography to contrast and compare HAI and neutralization data sets in naïve and preimmune ferrets and humans following natural infection and/or vaccination. Previously (13), female Fitch ferrets were infected with sublethal doses of one of three subtypes of influenza viruses (H1N1, H2N3, or H3N2) singly or in combination to mimic the primary infection heterogeneity seen in humans (Fig. 1). The ferrets were infected with WT influenza viruses isolated from humans, with the exception of the H2N3 influenza virus strain, which was isolated from chickens in 2004 (chicken/PA/04) (13).

These ferrets were termed “preimmune” and separated into six groups (Fig. 1). Each preimmune group had 20 ferrets, which were further divided into five groups for vaccination (*n* = 4 per vaccine group). The ferrets were allowed to recover for 60 days after each infection and then for another 60 days before vaccination (e.g., 120 dpi). The establishment of these preimmune groups would most closely reflect individuals born in the 1980s–1990s based on the virus strains used to establish preimmune conditions. We confirmed that ferrets had seroconverted to the homologous strains used to establish preimmunity (Fig. S1). Only ferrets infected with H2N3 had Ab titers to any of the antigens in our H2 panel prior to H2 vaccinations (Fig. S2). The ferrets were then divided into five groups and vaccinated intramuscularly twice, 28 days apart, with 15 μg of rH2 protein (e.g., Mal/Neth/01, Mal/Wisc/08, Computationally Optimized Broadly Reactive Antigen [COBRA] Z1, COBRA Z5, mock [no antigen]) mixed with MF59 adjuvant (Fig. 1). COBRA Z1 and Z5 antigens are candidate broadly cross-reactive antigens that were previously designed for increased breadth and immune performance (18). VLPs expressing the 12 different WT H2 HAs were used for the HAI assay, and live viruses were used for neutralization assays. H2 HA sequences used for VLPs and live H2Nx viruses used in our panels were selected based on phylogenetic diversity across all available H2 HA sequences, as previously described (18) (Fig. S3). NA antibodies elicited by the H1N1, H2N3, and H3N2 viral infections did not contribute to HAI titers (due to assay having HA Ab specificity) and are unlikely to have contributed to neutralization titers due to mock-vaccinated ferrets having neutralization titers at or below the limit of detection.

After infection, sera collected from ferrets infected with the H2N3 viruses elicited antibodies with robust HAI responses against the H2 VLP panel, whereas ferrets singly infected with the H1N1 or H3N2 influenza viruses had sporadic and lower-level HAI titers against the H2 VLPs (Table 1). After primary H2 vaccination, all vaccinated H2N3-preimmune ferrets had an average geometric mean HAI titer ≥1:40 against all VLPs in the panel, representing a ≥4-fold increase in HAI titer. While both the H1N1 and H3N2 preimmune, mock-vaccinated ferrets had geometric mean HAI titers ≤1:5 against the entire H2 VLP panel, the H2N3 preimmune, mock-vaccinated ferrets had serum HAI titers ≥1:40 against 7 of the 12 strains (Table 1). Compared with COBRA Z5 and the homotypic H2N3-infecting strains, COBRA Z1-vaccinated animals had nearly equivalent or higher HAI titers against most of the H2 VLPs assessed in our panel.

**TABLE 1:** Ferret HAIs after single vaccination^*a*^

Preimmunity	Sera	Mal/NL/01	Chk/Pots/84	Musk/Rus/14	Duk/Cam/13	J/57	Mosc/65	T/64	Duk/HK/78	Mal/WI/08	Sw/MO/06	Qu/RI/16	Tur/CA/08
H1N1	Mal/NL/01	4.30 (±1.74)	2.78 (±1.23)	4.12 (±1.94)	2.32 (±1.00)	2.97 (±1.35)	5.39 (±1.57)	3.26 (±1.60)	2.71 (±1.36)	4.55 (±1.11)	4.39 (±1.39)	5.93 (±1.53)	5.01 (±1.20)
H1N1	Mal/WI/08	4.97 (±1.79)	4.59 (±1.70)	4.66 (±1.85)	2.32 (±1.00)	3.81 (±1.52)	6.98 (±1.21)	4.52 (±1.57)	3.26 (±1.60)	5.55 (±1.45)	5.55 (±1.45)	5.81 (±1.88)	5.98 (±1.50)
H1N1	Z1 COBRA	3.33 (±1.53)	3.65 (±1.43)	2.32 (±1.00)	2.32 (±1.00)	3.17 (±1.43)	6.39 (±1.33)	3.24 (±1.29)	3.17 (±1.43)	4.29 (±1.55)	4.35 (±1.72)	3.97 (±1.87)	4.48 (±1.60)
H1N1	Z5 COBRA	2.54 (±1.20)	2.78 (±1.23)	2.32 (±1.00)	2.32 (±1.00)	3.26 (±1.60)	4.52 (±1.57)	2.78 (±1.23)	2.54 (±1.20)	3.42 (±1.41)	3.42 (±1.41)	2.86 (±1.51)	3.65 (±1.43)
H1N1	Mock	2.32 (±1.00)	2.32 (±1.00)	2.32 (±1.00)	2.32 (±1.00)	2.32 (±1.00)	2.32 (±1.00)	2.32 (±1.00)	2.32 (±1.00)	2.32 (±1.00)	2.32 (±1.00)	2.32 (±1.00)	2.32 (±1.00)
H2N3	Mal/NL/01	6.57 (±2.01)	6.76 (±2.04)	5.49 (±1.78)	10.24 (±1.16)	6.18 (±1.95)	6.76 (±2.04)	5.65 (±1.83)	6.46 (±2.04)	7.62 (±2.21)	7.78 (±2.24)	6.72 (±2.05)	7.78 (±2.24)
H2N3	Mal/WI/08	9.47 (±1.19)	10.32 (±1.00)	7.21 (±1.23)	4.69 (±1.81)	8.46 (±1.21)	10.01 (±1.14)	8.53 (±1.12)	8.78 (±1.13)	11.25 (±1.14)	11.02 (±1.13)	9.78 (±1.11)	11.56 (±1.04)
H2N3	Z1 COBRA	9.76 (±1.14)	9.76 (±1.14)	6.95 (±1.25)	10.04 (±1.10)	8.78 (±1.12)	10.01 (±1.14)	8.26 (±1.15)	9.00 (±1.16)	11.02 (±1.13)	10.78 (±1.10)	10.30 (±1.08)	11.06 (±1.05)
H2N3	Z5 COBRA	7.52 (±1.14)	9.06 (±1.06)	6.32 (±1.00)	7.63 (±1.30)	7.25 (±1.17)	9.04 (±1.11)	7.06 (±1.08)	7.25 (±1.17)	9.29 (±1.09)	9.53 (±1.11)	8.22 (±1.20)	9.27 (±1.13)
H2N3	Mock	5.27 (±1.17)	5.76 (±1.17)	2.32 (±1.00)	2.32 (±1.00)	6.56 (±1.08)	6.56 (±1.08)	2.32 (±1.00)	4.55 (±1.11)	2.86 (±1.51)	7.06 (±1.08)	6.56 (±1.08)	6.06 (±1.09)
H3N2	Mal/NL/01	2.32 (±1.00)	2.32 (±1.00)	2.32 (±1.00)	3.12 (±1.48)	2.32 (±1.00)	2.86 (±1.51)	2.32 (±1.00)	2.32 (±1.00)	2.32 (±1.00)	2.78 (±1.23)	2.32 (±1.00)	2.54 (±1.20)
H3N2	Mal/WI/08	2.32 (±1.00)	2.32 (±1.00)	2.32 (±1.00)	2.54 (±1.20)	2.32 (±1.00)	3.12 (±1.48)	2.32 (±1.00)	2.32 (±1.00)	2.32 (±1.00)	2.78 (±1.23)	2.32 (±1.00)	2.53 (±1.20)
H3N2	Z1 COBRA	3.12 (±1.48)	3.46 (±1.34)	2.32 (±1.00)	2.54 (±1.20)	2.78 (±1.23)	5.96 (±1.25)	2.32 (±1.00)	2.32 (±1.00)	4.11 (±1.48)	4.94 (±1.31)	4.52 (±1.57)	4.94 (±1.32)
H3N2	Z5 COBRA	2.32 (±1.00)	3.24 (±1.29)	2.32 (±1.00)	2.32 (±1.00)	2.54 (±1.20)	4.69 (±1.32)	2.32 (±1.00)	2.32 (±1.00)	3.24 (±1.29)	3.65 (±1.43)	3.12 (±1.48)	3.65 (±1.43)
H3N2	Mock	2.54 (±1.20)	2.32 (±1.00)	2.32 (±1.00)	2.32 (±1.00)	2.32 (±1.00)	2.32 (±1.00)	2.32 (±1.00)	2.32 (±1.00)	2.32 (±1.00)	2.54 (±1.20)	2.32 (±1.00)	2.32 (±1.00)
None	Mal/NL/01	2.32 (±1.00)	2.32 (±1.00)	2.32 (±1.00)	2.32 (±1.00)	2.32 (±1.00)	2.32 (±1.00)	2.32 (±1.00)	2.32 (±1.00)	2.32 (±1.00)	2.32 (±1.00)	2.32 (±1.00)	2.32 (±1.00)
None	Mal/WI/08	2.32 (±1.00)	2.32 (±1.00)	2.32 (±1.00)	2.32 (±1.00)	2.32 (±1.00)	2.32 (±1.00)	2.32 (±1.00)	2.32 (±1.00)	2.32 (±1.00)	2.32 (±1.00)	2.32 (±1.00)	2.32 (±1.00)
None	Z1 COBRA	2.71 (±1.36)	2.71 (±1.36)	2.32 (±1.00)	2.32 (±1.00)	2.98 (±1.65)	2.98 (±1.65)	2.32 (±1.00)	2.32 (±1.00)	2.32 (±1.00)	2.32 (±1.00)	4.29 (±1.55)	2.71 (±1.36)
None	Z5 COBRA	2.32 (±1.00)	2.32 (±1.00)	2.32 (±1.00)	2.32 (±1.00)	2.32 (±1.00)	2.32 (±1.00)	2.32 (±1.00)	2.32 (±1.00)	2.32 (±1.00)	2.32 (±1.00)	2.32 (±1.00)	2.32 (±1.00)
None	Mock	2.32 (±1.00)	2.32 (±1.00)	2.32 (±1.00)	2.32 (±1.00)	2.32 (±1.00)	2.32 (±1.00)	2.32 (±1.00)	2.32 (±1.00)	2.32 (±1.00)	2.32 (±1.00)	2.32 (±1.00)	2.32 (±1.00)
H1N1, H3N2	Mal/NL/01	4.82 (±1.73)	5.07 (±1.72)	3.61 (±1.74)	4.13 (±2.00)	3.26 (±1.60)	4.26 (±2.00)	3.12 (±1.48)	3.48 (±1.64)	3.27 (±1.49)	4.22 (±2.03)	4.79 (±1.74)	4.98 (±1.77)
H1N1, H3N2	Mal/WI/08	6.61 (±1.52)	7.42 (±1.43)	4.60 (±2.25)	7.83 (±1.32)	4.45 (±1.62)	7.85 (±1.32)	3.55 (±1.14)	5.29 (±1.45)	7.41 (±1.26)	8.24 (±1.17)	7.33 (±1.34)	8.03 (±1.12)
H1N1, H3N2	Z1 COBRA	5.53 (±1.64)	7.10 (±1.32)	4.41 (±1.76)	6.97 (±1.60)	5.27 (±1.17)	9.51 (±1.14)	5.05 (±1.11)	6.21 (±1.23)	7.41 (±1.26)	8.29 (±1.10)	7.74 (±1.18)	8.29 (±1.10)
H1N1, H3N2	Z5 COBRA	3.52 (±1.61)	5.51 (±1.20)	3.04 (±1.20)	5.37 (±1.98)	3.34 (±1.52)	6.95 (±1.25)	3.24 (±1.29)	3.84 (±1.50)	5.23 (±1.25)	6.45 (±1.26)	5.27 (±1.48)	6.24 (±1.20)
H1N1, H3N2	Mock	2.32 (±1.00)	2.32 (±1.00)	2.32 (±1.00)	2.32 (±1.00)	2.32 (±1.00)	2.32 (±1.00)	2.32 (±1.00)	2.32 (±1.00)	2.32 (±1.00)	2.32 (±1.00)	2.32 (±1.00)	2.32 (±1.00)
H3N2, H1N1	Mal/NL/01	2.32 (±1.00)	2.32 (±1.00)	2.32 (±1.00)	2.86 (±1.51)	2.54 (±1.20)	2.32 (±1.00)	2.32 (±1.00)	2.32 (±1.00)	2.32 (±1.00)	2.32 (±1.00)	2.32 (±1.00)	2.32 (±1.00)
H3N2, H1N1	Mal/WI/08	2.32 (±1.00)	2.32 (±1.00)	2.32 (±1.00)	4.89 (±1.67)	2.54 (±1.20)	2.32 (±1.00)	2.32 (±1.00)	2.32 (±1.00)	2.32 (±1.00)	2.32 (±1.00)	2.32 (±1.00)	2.32 (±1.00)
H3N2, H1N1	Z1 COBRA	2.32 (±1.00)	2.32 (±1.00)	2.32 (±1.00)	2.32 (±1.00)	2.54 (±1.20)	2.32 (±1.00)	2.32 (±1.00)	2.32 (±1.00)	2.32 (±1.00)	2.32 (±1.00)	2.32 (±1.00)	2.32 (±1.00)
H3N2, H1N1	Z5 COBRA	2.32 (±1.00)	2.32 (±1.00)	2.32 (±1.00)	4.52 (±1.57)	2.54 (±1.20)	2.32 (±1.00)	2.32 (±1.00)	2.32 (±1.00)	2.32 (±1.00)	2.32 (±1.00)	2.32 (±1.00)	2.32 (±1.00)
H3N2, H1N1	Mock	2.32 (±1.00)	2.32 (±1.00)	2.32 (±1.00)	2.32 (±1.00)	2.32 (±1.00)	2.32 (±1.00)	2.32 (±1.00)	2.32 (±1.00)	2.32 (±1.00)	2.32 (±1.00)	2.32 (±1.00)	2.32 (±1.00)

^*a*^ The geometric mean HAI titers (log2) for ferrets within a vaccine group (*n* = 4/vaccine/group) against 12 H2 VLPs are shown. Standard deviations for each data point are shown in parentheses. Sera from each ferret were obtained approximately 14 days post-primary vaccination. Column 1 has the preimmunity that each group of ferrets received, while column 2 has the vaccine that each group of ferrets received (*n* = 4/vaccine group). The geometric mean HAI titer is recorded for each of the 12 H2 VLPs in subsequent columns. Separate groups are indicated by shading.

Excluding the mock-vaccinated ferrets, each of the H1N1 preimmune ferret vaccine groups had similar or higher geometric mean HAI titers for each H2 antigen in our panel than the H3N2 preimmune ferrets. Ferrets preimmune to the H3N2 influenza virus had low HAI titers against each of the H2 VLPs in the panel, and no ferret had a >4-fold increase in HAI activity against the H2 influenza viruses compared to mock controls after vaccination (HAI ≤ 5). However, both COBRA Z1 and Z5 vaccinated ferrets had a >4-fold increase in HAI titer against 2–4 of the 12 H2 VLPs in the panel (Table 1). While none of the mock-vaccinated ferrets had detectable HAI titers against any H2 VLP, two ferrets had pr-immune titers to H1N1 influenza virus (geometric mean HAI titer ≥1:40 against the Mosc/65 VLP), and 11/12 ferrets had a ≥4-fold increase in HAI titers against the Mal/WI/08 and 8/12 against the Mal/NL/01 over background (Table 1). HAI titers in the Z1 and Z5 COBRA vaccinated ferrets had lower responses than ferrets vaccinated with H2 Mal/WI/08 (5/12) and Mal/NL/01 (2/12) with a ≥4-fold increased HAI titer over mock controls (Table 1).

Additional groups of ferrets were primed with sequential infections of two influenza viruses, either H1N1 followed by H3N2 or H3N2 followed by H1N1. The mock-vaccinated ferrets had HAI titers of ≤1:5 across the H2 panel in both of the double-infected, preimmune groups. Across all H2 vaccine groups, ferrets that were preimmune after H1N1 followed by H3N2 influenza virus infections demonstrated a geometric mean HAI >1:40 against 8 to 11 of the 12 VLPs in our panel. While HAI titers elicited by the Z5 COBRA rHA vaccine were generally less robust across the HAI panel, ferrets vaccinated with the Z1 COBRA HA vaccine had a ≥4-fold increase in HAI titers against all 12 of the H2 VLPs (Table 1). In the H3N2 and H1N1 preimmune group, H2 HAI titers were lower across the panel compared to other preimmune groups. In fact, the Mal/WI/08 and Z5 COBRA vaccinated ferrets only had a ≥4fold increase in HAI titers against a single H2 VLP (Duke/Cam/13) (Table 1), showing the significance of influenza A virus infection order and immune imprinting on downstream immune responses to H2 antigens.

To effectively evaluate the statistical significance of each vaccine and preimmune group, a statistician was consulted. Using a one-way ANOVA with Tukey *post hoc* test, the Z1 COBRA vaccine had a significantly higher overall geometric mean HAI titer compared to the other vaccine groups, while the Mal/NL/01 vaccine had significantly lower overall geometric mean titers compared to the other vaccine groups (Fig. S4). There was no statistical difference between the Z5 COBRA and Mal/WI/08 vaccine groups (Fig. S4). Regarding preimmunity, the H2N2 preimmune ferrets had significantly higher geometric mean HAI titers compared to each of the other preimmune groups (Fig. S4). The geometric mean HAI titers for the H1N1, H3N2, and H1N1-H3N2 preimmune groups were not statistically different from each other but were all statistically higher than both the H3N2-H1N1 preimmune and mock preimmune groups (Fig. S4). The H3N2-H1N1 preimmune and mock preimmune groups were not statistically different from each other (Fig. S4).

### Antigenic relationships across the preimmune groups by cartography

**Fig 2:**
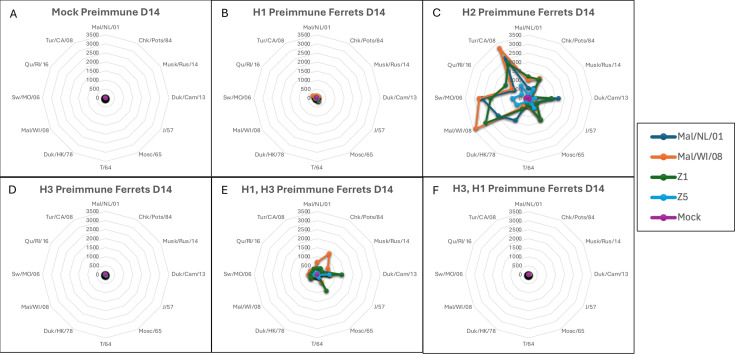
HAI radial graph post-prime H2 vaccination. Vaccine groups are color-coded per the legend and consistent between each graph. The circles in each graph represent the HAI titer. (**A**) Mock preimmune ferrets, (**B**) H1 preimmune ferrets, (**C**) H2 preimmune ferrets, (**D**) H3 preimmune ferrets, (**E**) H1, H3 preimmune ferrets, and (**F**) H3, H1 preimmune ferrets.

Radial graphs were generated to clearly visualize the impact of pre-exposure histories and vaccines on antigen recognition (Fig. 2). On D14, the H2 preimmune ferrets had the highest HAI titers and recognized the highest number of antigens of any preimmune group. The mock preimmune group had the lowest geometric mean HAI titers (Fig. 2), while the H3 and H1 preimmune ferrets only had HAI titers to Duk/Cam/13. Being infected with H3N2 alone or prior to an H1N1 infection dramatically impacted H2 vaccine performance in ferrets.

**Fig 3:**
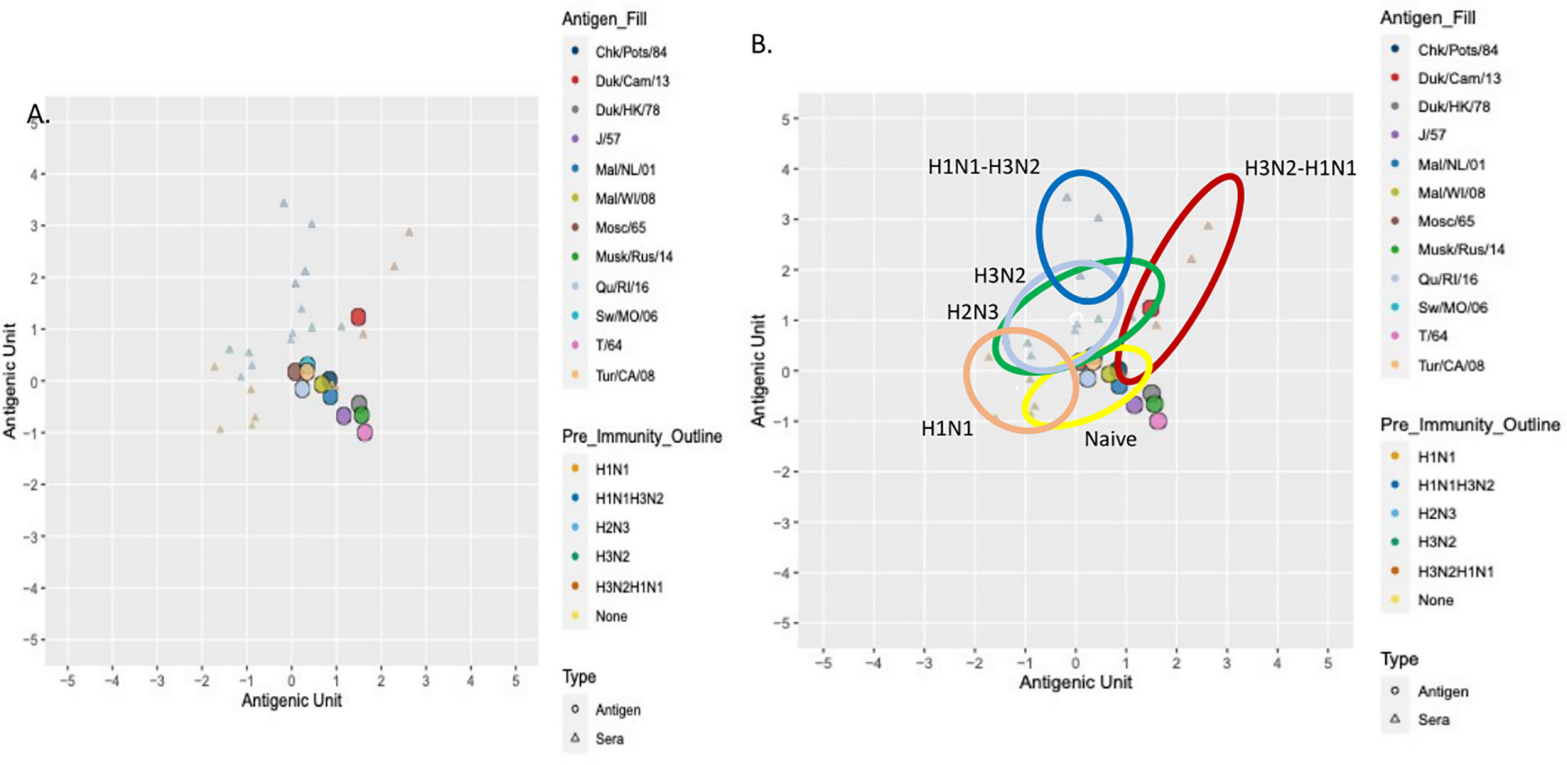
Ferret HAI antigenic cartography maps after prime vaccination. (**A**) Antigenic maps were generated for each ferret preimmune group in HAIs. Antigens are represented as circles, and sera from the ferrets are represented by triangles. (**B**) The sera from each influenza preimmune group are grouped within each colored circle for clarity.

Antigenic cartography was applied to further examine the HAI data following the first vaccinations or infections (Fig. 3). In antigenic cartography, antigens and sera that cluster in the center respond most similarly, while those that radiate outward are increasingly divergent from the other antigens/sera. Each of the antigens clustered together near the center of the graph, with the exception of the Duk/Cam/13 HA antigen (Fig. 3A and B). In general, the sera from each preimmune group clustered together near the center of the graph, which was especially evident in the H2N3 and H3N2 vaccinated ferrets. For the other preimmune groups, ferrets preimmune to the H1N1 influenza virus, and to a greater extent the H1N1-H3N2 and the H3N2-H1N1 preimmune ferrets, had a dramatically altered serological response with expanded HAI breadth against the H2 VLP panel (Fig. 3A and B). The serum samples collected from the four naïve preimmune groups all overlapped on the antigenic graph since there was no variability in their HAI titers (Fig. 3A and B).

### Serum HAI responses after secondary vaccinations

Preimmune ferrets were boosted with the same H2 antigen, and convalescent serum was isolated on day 56 post-boost (Fig. 1). After the second vaccination, the H2N3 preimmune ferret HAI titers did not drastically change compared to the HAI titers following the first vaccination (Tables 1 and 2). Post boost, in ferrets preimmune after H1N1 influenza virus infection, the H2 HAI titers not only increased slightly against the Mal/WI/08 and MAL/NL/01 VLPs, but a broader ≥4-fold increase in H2 HAI titers was noted against the other H2 strains. However, in the COBRA vaccine groups, the geometric mean H2 HAI titers increased ≥7- to 10-fold over preimmune vaccinated animals demonstrating their superiority as vaccine antigens compared to WT antigens (Table 2). In the non-preimmune group, each of the rH2 vaccinated ferrets showed increases in HAI titers after boost (Table 2). The Mal/NL/01 and Mal/WI/08 vaccination groups had geometric mean HAI titers >1:40, with fourfold elevated titers in 8/12 and 7/12 antigens, respectively (Table 2). The Z1 and Z5 COBRA vaccination groups had a geometric mean HAI >1:40 to 11 and 9 of the 12 of the VLPs in the panel, with 11/12 and 10/12 fourfold elevated titers over baseline, respectively (Table 2). In the H3N2 preimmune group, the Mal/NL/01 and Mal/WI/08 vaccination groups had a geometric mean HAI >1:40 to 7 and 11 of the 12 VLPs in the panel (Table 2). Both the Z1 and Z5 COBRA vaccine groups had a geometric mean HAI >1:40 to 10 of the 12 VLPs in the panel (Table 2). Overall, the H2N3 and H1N1 preimmune ferrets had higher HAI titers to the H2 VLPs than the H3N2 preimmune ferrets.

**TABLE 2:** Ferret HAI after second vaccinatione^*a*^

Preimmunity	Vaccine	Mal/NL/01	Chk/Pots/84	Musk/Rus/14	Duk/Cam/13	J/57	Mosc/65	T/64	Duk/HK/78	Mal/WI/08	Sw/MO/06	Qu/RI/16	Tur/CA/08
H1N1	Mal/NL/01	5.96 (±1.25)	5.80 (±1.1)	4.66 (±1.85)	2.32 (±1.00)	5.51 (±1.20)	8.06 (±1.07)	3.95 (±1.62)	5.01 (±1.20)	7.29 (±1.12)	7.29 (±1.12)	8.29 (±1.10)	8.03 (±1.12)
H1N1	Mal/WI/08	5.58 (±1.42)	4.26 (±1.21)	2.54 (±1.20)	2.32 (±1.00)	5.16 (±1.36)	7.74 (±1.18)	3.17 (±1.43)	4.73 (±1.27)	6.52 (±1.16)	6.28 (±1.14)	7.52 (±1.14)	7.52 (±1.14)
H1N1	Z1 COBRA	6.49 (±1.20)	5.52 (±1.47)	4.39 (±1.39)	2.32 (±1.00)	5.05 (±1.73)	7.85 (±1.52)	4.86 (±1.40)	5.05 (±1.73)	7.61 (±1.31)	6.57 (±1.38)	8.20 (±1.22)	7.61 (±1.31)
H1N1	Z5 COBRA	6.12 (±1.35)	6.02 (±1.17)	4.18 (±1.58)	2.32 (±1.00)	5.38 (±1.38)	7.77 (±1.15)	6.63 (±1.33)	4.94 (±1.32)	7.52 (±1.14)	7.42 (±1.27)	7.83 (±1.34)	7.99 (±1.18)
H1N1	Mock	2.32 (±1.00)	2.32 (±1.00)	2.32 (±1.00)	2.32 (±1.00)	2.32 (±1.00)	2.32 (±1.00)	2.32 (±1.00)	2.32 (±1.00)	2.32 (±1.00)	2.32 (±1.00)	2.32 (±1.00)	2.32 (±1.00)
H2N3	Mal/NL/01	6.36 (±1.99)	5.86 (±1.86)	5.30 (±1.74)	6.36 (±1.99)	5.81 (±1.88)	6.57 (±2.01)	5.49 (±1.78)	5.65 (±1.83)	7.09 (±2.11)	7.27 (±2.14)	6.03 (±1.90)	7.09 (±2.11)
H2N3	Mal/WI/08	8.86 (±1.30)	8.81 (±1.07)	6.80 (±1.09)	8.51 (±1.15)	7.96 (±1.21)	9.27 (±1.13)	7.52 (±1.14)	7.52 (±1.14)	10.24 (±1.16)	11.02 (±1.13)	8.98 (±1.18)	10.54 (±1.10)
H2N3	Z1 COBRA	9.97 (±1.18)	9.53 (±1.11)	7.56 (±1.07)	9.97 (±1.18)	8.53 (±1.12)	10.56 (±1.05)	7.96 (±1.21)	8.53 (±1.12)	10.78 (±1.10)	10.78 (±1.10)	9.78 (±1.11)	10.78 (±1.10)
H2N3	Z5 COBRA	7.99 (±1.18)	8.81 (±1.07)	6.80 (±1.09)	7.77 (±1.15)	6.73 (±1.21)	8.29 (±1.10)	6.80 (±1.09)	7.02 (±1.14)	9.29 (±1.09)	8.98 (±1.18)	8.22 (±1.20)	9.06 (±1.06)
H2N3	Mock	5.27 (±1.17)	5.76 (±1.17)	2.32 (±1.00)	2.32 (±1.00)	6.56 (±1.08)	6.56 (±1.08)	2.32 (±1.00)	4.55 (±1.10)	2.86 (±1.51)	7.06 (±1.08)	6.56 (±1.08)	6.06 (±1.09)
H3N2	Mal/NL/01	3.48 (±1.63)	4.52 (±1.57)	2.32 (±1.00)	5.85 (±2.12)	3.12 (±1.48)	5.24 (±1.78)	2.32 (±1.00	3.57 (±1.52)	4.22 (±1.60)	4.22 (±1.60)	4.45 (±1.62)	4.45 (±1.62)
H3N2	Mal/WI/08	6.06 (±1.09)	6.06 (±1.09)	2.32 (±1.00)	5.61 (±2.28)	5.56 (±1.09)	8.06 (±1.07)	5.27 (±1.17)	5.56 (±1.09)	7.50 (±1.17)	8.29 (±1.10)	7.29 (±1.11)	8.29 (±1.10)
H3N2	Z1 COBRA	5.96 (±1.25)	6.76 (±1.17)	2.97 (±1.35)	4.85 (±2.38)	5.58 (±1.42)	7.99 (±1.18)	3.65 (±1.43)	5.35 (±1.41)	7.74 (±1.18)	8.75 (±1.16)	7.99 (±1.18)	9.00 (±1.16)
H3N2	Z5 COBRA	6.19 (±1.28)	6.28 (±1.14)	2.54 (±1.20)	6.65 (±2.08)	5.96 (±1.25)	8.29 (±1.10)	2.71 (±1.36)	5.96 (±1.25)	7.29 (±1.12)	8.03 (±1.12)	7.74 (±1.18)	7.99 (±1.18)
H3N2	Mock	2.32 (±1.00)	2.32 (±1.00)	2.32 (±1.00)	2.32 (±1.00)	2.32 (±1.00)	2.32 (±1.00)	2.32 (±1.00)	2.32 (±1.00)	2.32 (±1.00)	2.32 (±1.00)	2.32 (±1.00)	2.32 (±1.00)
Mock	Mal/NL/01	4.55 (±1.11)	4.55 (±1.11)	2.32 (±1.00)	3.67 (±1.70)	2.98 (±1.65)	4.12 (±1.94)	2.32 (±1.00)	2.32 (±1.00)	2.32 (±1.00)	3.97 (±1.87)	6.02 (±1.167)	4.26 (±1.21)
Mock	Mal/WI/08	4.29 (±1.55)	4.11 (±1.48)	2.32 (±1.00)	2.32 (±1.00)	4.12 (±1.94)	4.40 (±2.09)	2.32 (±1.00)	2.32 (±1.00)	2.32 (±1.00)	3.93 (±1.89)	5.11 (±1.70)	4.52 (±1.57)
Mock	Z1 COBRA	6.92 (±1.27)	7.02 (±1.14)	2.32 (±1.00)	8.56 (±1.06)	8.75 (±1.16)	8.75 (±1.16)	3.09 (±1.16)	5.27 (±1.75)	8.03 (±1.12)	9.04 (±1.11)	7.99 (±1.18)	7.21 (±1.23)
Mock	Z5 COBRA	3.61 (±1.74)	4.64 (±1.67)	2.32 (±1.00)	3.19 (±1.89(	5.84 (±1.87)	5.47 (±1.79)	2.32 (±1.00)	2.98 (±1.65)	3.09 (±1.78)	5.47 (±1.79)	5.90 (±1.31)	5.47 (±1.24)
Mock	Mock	2.32 (±1.00)	2.32 (±1.00)	2.32 (±1.00)	2.32 (±1.00)	2.32 (±1.00)	2.32 (±1.00)	2.32 (±1.00)	2.32 (±1.00)	2.32 (±1.00)	2.32 (±1.00)	2.32 (±1.00)	2.32 (±1.00)
H1N1, H3N2	Mal/NL/01	6.33 (±1.37)	5.05 (±1.66)	4.30 (±1.74)	4.04 (±1.97)	3.61 (±1.74)	4.52 (±2.16)	3.48 (±1.64)	3.61 (±1.74)	4.22 (±2.03)	4.49 (±2.17)	5.33 (±1.86)	4.22 (±2.03)
H1N1, H3N2	Mal/WI/08	8.24 (±1.18)	7.69 (±1.23)	5.81 (±1.41)	8.88 (±1.28)	5.96 (±1.25)	8.20 (±1.22)	4.49 (±1.25)	5.12 (±1.38)	8.24 (±1.18)	9.44 (±1.20)	8.89 (±1.25)	7.98 (±1.19)
H1N1, H3N2	Z1 COBRA	8.06 (±1.07)	8.03 (±1.12)	5.90 (±1.32)	8.06 (±1.07)	6.80 (±1.09)	9.56 (±1.05)	5.80 (±1.10)	6.56 (±1.08)	8.03 (±1.12)	9.56 (±1.05)	8.53 (±1.12)	8.06 (±1.07)
H1N1, H3N2	Z5 COBRA	6.49 (±1.20)	6.24 (±1.20)	4.55 (±1.11)	4.12 (±1.94)	5.51 (±1.20)	7.52 (±1.14)	3.99 (±1.26)	4.69 (±1.32)	6.49 (±1.20)	6.95 (±1.25)	6.73 (±1.21)	6.52 (±1.16)
H1N1, H3N2	Mock	2.32 (±1.00)	2.32 (±1.00)	2.32 (±1.00)	2.32 (±1.00)	2.32 (±1.00)	2.32 (±1.00)	2.32 (±1.00)	2.32 (±1.00)	2.32 (±1.00)	2.32 (±1.00)	2.32 (±1.00)	2.32 (±1.00)
H3N2, H1N1	Mal/NL/01	4.01 (±1.58)	4.12 (±1.94)	5.65 (±1.83)	8.75 (±1.16)	7.68 (±1.25)	3.19 (±1.89)	4.89 (±1.67)	2.32 (±1.00)	3.81 (±1.53)	5.42 (±1.81)	3.81 (±1.80)	4.68 (±1.63)
H3N2, H1N1	Mal/WI/08	3.90 (±1.43)	4.30 (±1.74)	5.84 (±1.87)	8.43 (±1.23)	7.21 (±1.23)	3.61 (±1.74)	4.52 (±1.57)	2.32 (±1.00)	3.97 (±1.87)	7.74 (±1.18)	4.49 (±1.79)	5.47 (±1.79)
H3N2, H1N1	Z1 COBRA	3.84 (±1.50)	3.67 (±1.70)	7.44 (±1.25)	8.75 (±1.16)	7.41 (±1.27)	3.26 (±1.60)	4.19 (±1.64)	2.32 (±1.00)	3.26 (±1.60)	7.66 (±1.28)	2.54 (±1.20)	4.82 (±1.73)
H3N2, H1N1	Z5 COBRA	3.90 (±1.43)	4.33 (±1.51)	6.19 (±1.28)	7.41 (±1.27)	5.13 (±1.85)	4.11 (±1.48)	4.11 (±1.48)	2.32 (±1.00)	4.49 (±1.25)	6.19 (±1.28)	3.67 (±1.70)	5.51 (±1.20)
H3N2, H1N1	Mock	2.32 (±1.00)	2.32 (±1.00)	2.32 (±1.00)	2.32 (±1.00)	2.32 (±1.00)	2.32 (±1.00)	2.32 (±1.00)	2.32 (±1.00)	2.32 (±1.00)	2.32 (±1.00)	2.32 (±1.00)	2.32 (±1.00)

^*a*^ The geometric mean HAI titers (log2) for ferrets within a vaccine group (*n* = 4/vaccine/group) against 12 H2 VLPs is shown. Standard deviations for each data point are shown in parentheses. Sera from each ferret were obtained approximately 14 days post-secondary vaccination. Column 1 has the preimmunity that each group of ferrets received, while column 2 has the vaccine that each group of ferrets received (*n* = 4/vaccine group). The geometric mean HAI titer is recorded for each of the 12 H2 VLPs in subsequent columns. Separate groups are delineated by shading.

In the H1N1, H3N2 preimmune group, animals responded more efficiently against the H2 virus after vaccine boost than similarly boosted H1N1 and H3N2 preimmune groups alone. For example, H1N1, H3N2 preimmune animals mounted ≥4-fold increased H2 HAI responses to 11/12 H2 VLPs in Mal/NL/01 and Mal/WI/08 vaccination groups, with geometric mean titers of ~1/129 and 1/482, respectively. Again, the Z1 COBRA vaccine proved superior, as ≥4-fold increased H2 VLP titers were evident over mocks, and increased geometric mean titers of 1/425 over 1/129, respectively. Both titers were superior to Z5 COBRA responses as well (Table 2). Surprisingly, the H3N2, H1N1 preimmune group had attenuated overall H2 HAI titers across all vaccine groups as compared with H1N1, H3N2 groups. For example, the Mal/WI/08 and Z5 COBRA groups showed a ≥4-fold increased HAI response over mocks, against a single H2 VLP (Table 2). strain vaccination groups had a geometric mean HAI >1:40 against 9 and 10 of the 12 VLPs in the panel, respectively (Table 2). However, both the Z1 COBRA and Z5 COBRA vaccine groups had a geometric mean HAI >1:40 to 6 and 5 of the 12 VLPs in the panel, respectively (Table 2). It is noteworthy that H3N1, H1N1 preimmune animals mounted minimal responses against Duk/HK/78.

### Radial graphs and cartography after H2 vaccine boost

**Fig 4:**
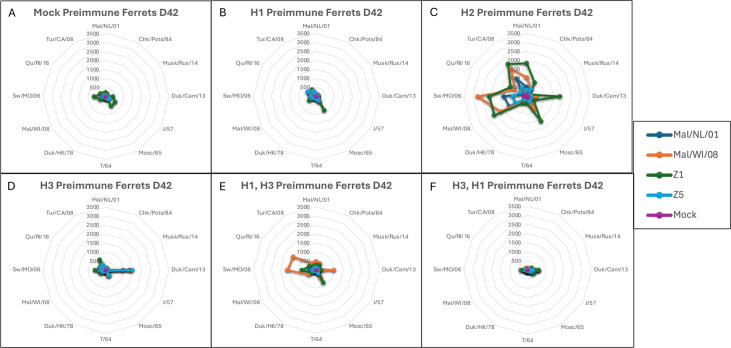
HAI radial graph post-prime H2 vaccination. Vaccine groups are color-coded per the legend and are consistent between each graph. The circles in each graph represent the HAI titer. (**A**) Mock preimmune ferrets, (**B**) H1 preimmune ferrets, (**C**) H2 preimmune ferrets, (**D**) H3 preimmune ferrets, (**E**) H1, H3 preimmune ferrets, and (**F**) H3, H1 preimmune ferrets.

Radial graphs were generated to visualize the HAI responses in the preimmune ferrets after H2 boost (Fig. 4). Overall, the HAI titers increased to all antigens except for the Mal/WI/08 vaccinated, H1, H3 preimmune ferrets, who showed a dramatic decrease in HAI titers to the Chk/Pots/84 antigen (Fig. 4). The H3, H1 preimmune ferret and the H3 preimmune ferret HAI responses predominantly increased only to Duk/Cam/13 and Duk/Cam/13, respectively (Fig. 4).

**Fig 5:**
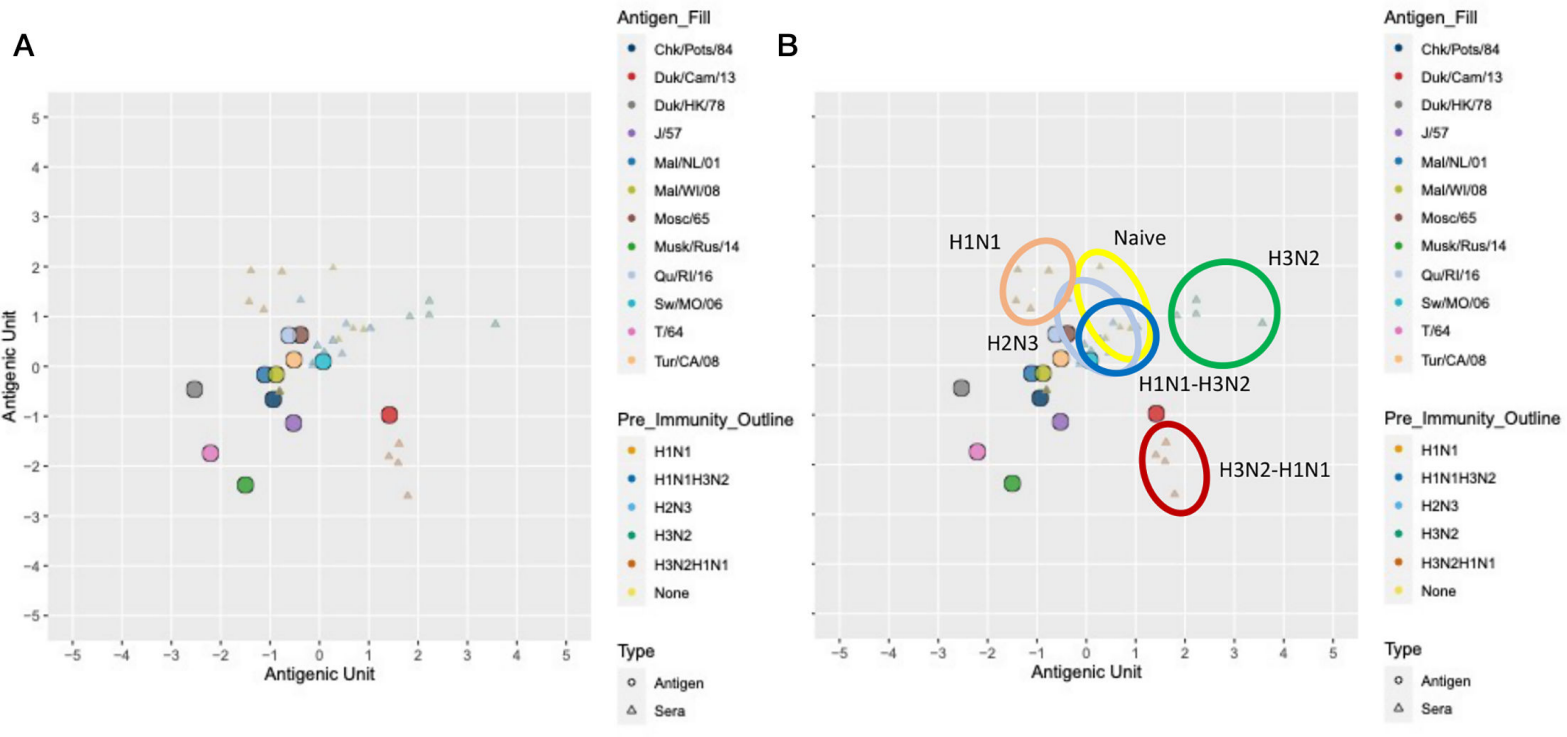
Ferret HAI antigenic cartography maps after boost vaccination. (**A**) Antigenic maps were generated for each ferret preimmune group in HAIs. Antigens are represented as circles and sera from the ferrets are represented by triangles. The preimmune groups are beginning to separate from each other and the antigens due to increased HAI titer differences compared with prime vaccinations. (**B**) The sera from each influenza preimmune group are grouped within each colored circle for clarity.

Antigenic cartography was used to analyze HAI activity of ferret serum samples following the second vaccination (Fig. 5). The HA antigens Musk/Rus/14, Duk/Cam/13, T/64, and Duk/HK/78 were antigenically farthest away from the other HA antigens (Fig. 5). Sera from the preimmune groups separated from each other (Fig. 5). All the serum samples collected from the naïve ferrets, as well as ferrets preimmune to H2N3, H1N1-H3N2, or H3N2-H1N1 influenza viruses, were clustered closely together on the antigenic map with the other serum samples within their same influenza group (Fig. 5). Each of the preimmune animals elicited HAI responses that grouped tightly together with the other animals in its preimmune group. The H3N2 and H3N2-H1N1 preimmune ferrets had sera with the furthest antigenic distance from the other preimmune groups (Fig. 5). Interestingly, first exposure to H1N1 appeared to elicit imprinted responses that were more favorable to H2N2 vaccination, as compared to H3N2 first exposure.

Based on the antigenic cartography, Musk/Rus/14, T/64, and Duk/HK/78 sera were separated from the other sera in the HAI antigenic map (Fig. 5). T/64 has mutations at sites 132, 139, and 188. Linster et al. found that site 139 (asparagine [N] to lysine [K]) had the most substantial antigenic change of any of the single amino acid mutations (5) (Fig. S5). Both T/64 and Musk/Rus/14 had the N-to-K mutation, which is likely driving their divergence from the other sera and antigens, respectively (Fig. S5). Mutations at sites 132 and 184 both had minor changes in antigenic distance individually. Both Musk/Rus/14 and T/64 have a glutamine (Q) and methionine (M), respectively, at site 132, while all the other viruses (except Chik/Pots/84) encode arginine (R) (Fig. S5). Additionally, both Musk/Rus/14 and T/64 have a positively charged lysine (K) at site 139, while all other viruses encode an asparagine (N) (Fig. S6). Of the sites identified by Linster et al., Duk/HK/78 did not have a mutation in any site (5) (Fig. S5). These data suggest that other amino acid residue substitutions are also contributing to the antigenic variation of Musk/Rus/14, T/64, and Duk/HK/78.

Additionally, Musk/Rus/14 has a unique mutation at site 140, which was not found by Linster et al. (5) (Fig. S6). This proline (P) to serine (S) mutation at site 140, along with the mutations at sites 132 and 139, are all likely contributing to the low HAI titers against Musk/Rus/14. Both T/64 and Duk/HK/78 also had mutations at site 151, which was not reported by Linster et al. (5) (Fig. S6). T/64 encodes a glutamic acid (E), while Duk/HK/78 encodes a threonine (T) at site 151, while all other H2 HA encode a lysine (K) at site 151. These mutations may contribute to the lower HAI titers against both T/64 and Duk/HK/78. In addition to site 151, Duk/HK/78 also has nine other amino acid changes that are not present in any of the other H2 influenza viruses.

**Fig 6:**
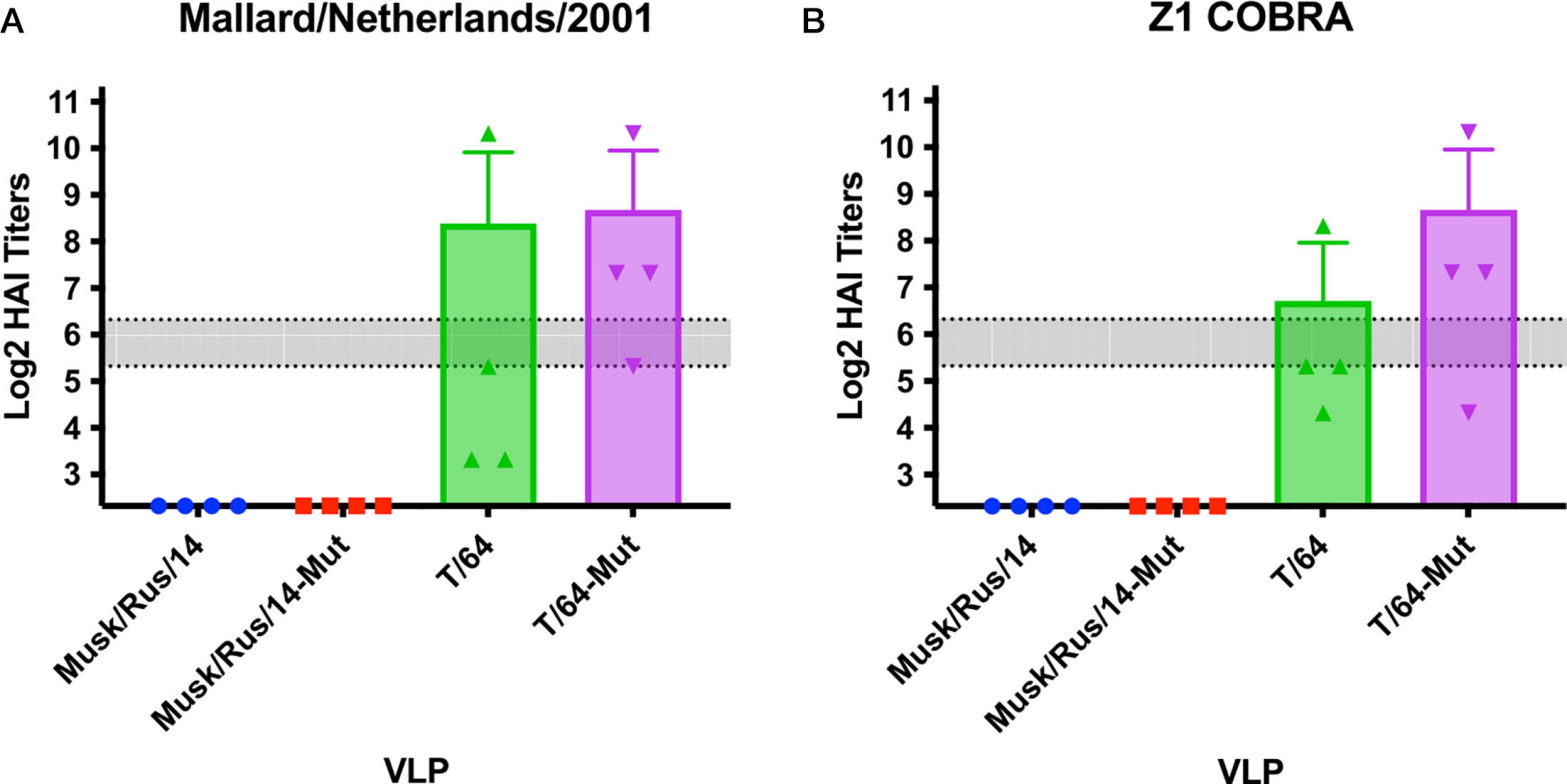
Antigenic significance of sites 140 and 151. BALB/c mice were vaccinated with 3 μg of recombinant proteins (Mal/NL/01 or Z1 COBRA) with MF50 oil-in-water emulsion adjuvant. (**A**) Mal/NL/01 vaccinated mice had HAI below the limit of detection for both the WT and mutant (S140P) Musk/Rus/14 VLPs. The geometric mean HAI titer against the T/64 mutant (E151K) increased but was not significant over the WT T/64 VLP. (**B**) Z1 COBRA vaccinated mice had HAI below the limit of detection for both the WT and mutant (S140P) Musk/Rus/14 VLPs. The geometric mean HAI titer against the T/64 mutant (E151K) increased but was not significant over the WT T/64 VLP.

To evaluate the significance of site 140 in Musk/Rus/14 and site 151 in T/64, we generated VLPs containing each mutant HA protein. We generated both the WT HA sequences of both Musk/Rus/14 and T/64 while also utilizing site-directed mutagenesis (SDM) to mutate site 140 in Musk/Rus/14 from serine(S) to proline (P) (S140P) and site 151 in T/64 from glutamic acid (E) to lysine (K) (E151K). We then vaccinated BALB/c mice with 3 μg of recombinant HA (rHA) with MF59 oil-water emulsion adjuvant. The mice (*n* = 4/group) were vaccinated twice, 4 weeks apart, before being bled 2 weeks post-boost. The antigens used for vaccinations were Z1 COBRA and Mal/NL/01 that we have used previously (18). The geometric mean HAI titers against T/64 did increase for both Mal/NL/01 and Z1 COBRA but did not achieve statistical significance (*P* = 0.1265 NL and *P* = 0.2709 Z1) (Fig. 6). The HAI titers against Musk/Rus/14 did not increase and remained below the limit of detection (Fig. 6).

### Live virus neutralization assays

Pooled sera collected from the ferrets after the second vaccination were used in classical neutralization assays against a panel of heterologous H2Nx influenza viruses. Neutralization assays quantify all antibodies that block viral attachment, replication, and spread. The mock-vaccinated ferrets all had neutralization titers <1:10 in all the preimmune groups apart from the H2N3 preimmune group (Table 3). In the H2N3 preimmune group, the mock-vaccinated ferrets had a geometric mean neutralization titers ≥1:160 to one of the seven H2 viruses in the panel.

**TABLE 3:** Ferret neutralization titers^*a*^

Preimmunity	Vaccine	Chk/Pots/84	Chk/PA/04	For/57	T/64	Duk/HK/78	Sw/MO/06	Mal/MN/08
H2N2	Mal/NL/01	9.32	8.57	5.58	5.09	9.32	9.32	8.82
H2N2	Mal/WI/08	9.32	9.07	5.58	5.81	9.32	9.32	9.32
H2N2	Z1 COBRA	9.32	9.32	7.07	6.07	9.32	9.32	9.32
H2N2	Z5 COBRA	8.82	9.32	5.32	8.57	9.07	9.32	8.32
H2N2	Mock	6.57	7.29	3.00	4.58	5.58	9.07	6.57
H3N2	Mal/NL/01	4.09	2.81	2.32	4.32	3.58	8.82	4.09
H3N2	Mal/WI/08	5.58	6.07	2.32	4.81	4.58	9.32	6.07
H3N2	Z1 COBRA	7.57	5.81	2.81	5.32	6.07	9.32	6.57
H3N2	Z5 COBRA	6.82	6.82	2.32	4.32	5.09	9.32	5.58
H3N2	Mock	2.32	2.81	2.32	2.32	2.32	2.58	2.58
H1N1	Mal/NL/01	6.82	4.32	2.32	6.57	4.81	9.07	4.58
H1N1	Mal/WI/08	6.82	5.32	2.58	9.07	5.09	9.32	7.82
H1N1	Z1 COBRA	9.07	8.07	4.32	6.32	8.07	9.32	8.07
H1N1	Z5 COBRA	7.07	5.58	3.32	4.32	6.32	9.32	3.58
H1N1	Mock	3.00	2.58	2.32	2.32	2.32	2.32	2.32
H3N2, H1N1	Mal/NL/01	8.07	7.32	4.81	5.09	6.57	9.32	7.07
H3N2, H1N1	Mal/WI/08	6.82	6.82	3.32	5.09	6.07	9.32	7.07
H3N2, H1N1	Z1 COBRA	9.32	8.57	4.81	7.32	9.07	9.32	7.82
H3N2, H1N1	Z5 COBRA	7.82	7.32	4.09	5.58	7.32	9.32	7.32
H3N2, H1N1	Mock	2.32	2.32	2.81	2.32	2.32	2.58	2.58
H1N1, H3N2	Mal/NL/01	9.32	9.32	5.09	2.32	6.32	9.32	9.32
H1N1, H3N2	Mal/WI/08	9.07	8.07	5.32	5.32	6.32	9.32	8.57
H1N1, H3N2	Z1 COBRA	7.82	9.32	6.07	6.82	6.57	9.32	9.07
H1N1, H3N2	Z5 COBRA	8.07	8.07	4.32	5.32	6.82	9.32	7.57
H1N1, H3N2	Mock	2.32	2.58	2.32	2.32	2.32	2.32	2.32
Mock	Mal/NL/01	5.09	3.81	2.32	3.32	3.32	9.32	4.32
Mock	Mal/WI/08	3.58	4.09	2.32	2.32	2.81	9.32	4.32
Mock	Z1 COBRA	9.07	7.57	5.32	6.32	7.82	9.32	6.32
Mock	Z5 COBRA	7.32	4.81	2.58	3.32	5.81	8.57	3.81
Mock	Mock	2.32	2.32	2.32	2.32	2.32	2.32	2.32

^*a*^ The geometric mean neutralization titers (log2) for ferrets within a vaccine group (*n* = 4/vaccine/group) against 7 H2 influenza viruses are shown. Sera from each ferret were obtained approximately 14 days post-secondary vaccination. Column 1 has the preimmunity that each group of ferrets received, while column 2 has the vaccine that each group of ferrets received (*n* = 4/vaccine group). The geometric mean neut titer is recorded for each of the seven H2 influenza viruses in subsequent columns. Separate groups are delineated by shading.

Preexposure to H2N3 viruses significantly boosted immune responses across the panel. The Mal/NL/01, Mal/WI/08, and Z1 COBRA vaccination groups all had a geometric mean fourfold elevated neutralization titers over baseline to 5/8 H2Nx viruses compared with 6/8 H2Nx viruses for Z1 COBRA. In general, neutralization titers ≥1:160 to five of the seven H2 viruses, while the Z5 COBRA vaccination group had a geometric mean neut titer ≥1:160 to six of the seven H2 viruses (Table 3). In the H1N1 preimmune group, neutralization titers were reduced as compared to preimmune H2N3 vaccinated groups, although the COBRA vaccines elicited fourfold increased neutralization titers against more H2 antigens in the panel. Both the Mal/NL/01 and Z5 COBRA vaccination groups had a geometric mean neut titer ≥1:160 to one of the seven H2 viruses while the Mal/WI/08 and Z1 COBRA vaccination groups had a geometric mean neut titer ≥1:160 to three and five of the seven H2 viruses, respectively (Table 3). In the H3N2 preimmune group, the Mal/NL/01, Mal/WI/08, and Z5 COBRA vaccination groups all had geometric mean neut titer ≥1:160 to one of the seven H2 viruses, while the Z1 COBRA vaccination group had a geometric mean neut titer ≥1:160 to two of the seven H2 viruses in the panel (Table 3).

In the H1N1, H3N2 preimmune group, the Mal/NL/01, Mal/WI/08, and Z1 COBRA and Z5 COBRA vaccination groups all had geometric mean neut titer ≥1:160 to four of the seven viruses in the panel (Table 3). In the H3N2, H1N1 preimmune group, the Mal/NL/01 vaccination group had a geometric mean neut titer ≥1:160 to three of the seven H2 viruses in the panel, while the Mal/WI/08 vaccination group had a geometric mean neut titer ≥1:160 to one of the seven H2 viruses in the panel. The Z1 and Z5 COBRA vaccination group had a geometric mean neut titer ≥1:160 to six and five of the seven H2 viruses in the panel, respectively (Table 3). In the non-preimmune group, each of the vaccination groups other than the mock vaccination group recognized the Sw/MO/06 virus had a geometric mean neut titer ≥1:160 (upper limit of detection). The Mal/NL/01 and Mal/WI/08 did not have a geometric mean neut titer ≥1:160 to any of the other six H2 viruses in the panel while the Z1 and Z5 COBRA vaccination groups had a geometric mean neut titer ≥1:160 to four and two of the other six H2 viruses in the panel, respectively (Table 3).

Overall, the H2N3 preimmune groups have the highest geometric mean neutralization titers across all seven viruses, while the H3N2 and mock preimmune groups had the lowest geometric mean neutralization titers across all seven viruses. Unsurprisingly, the H2N3 preimmune group had the highest geometric mean neutralization titers. However, the H1N1, H3N2 preimmune group also elicited superior neutralization titers as compared to the H3N2, H1N1 preimmune groups across all seven viruses with a geometric mean neut titer ≥1:220 for all of the non-mock-vaccinated groups. The Z1 COBRA vaccination group also had the highest geometric mean neut titer across all six preimmune vaccine groups.

### Antigenic cartography: live virus neutralization assays

**Fig 7:**
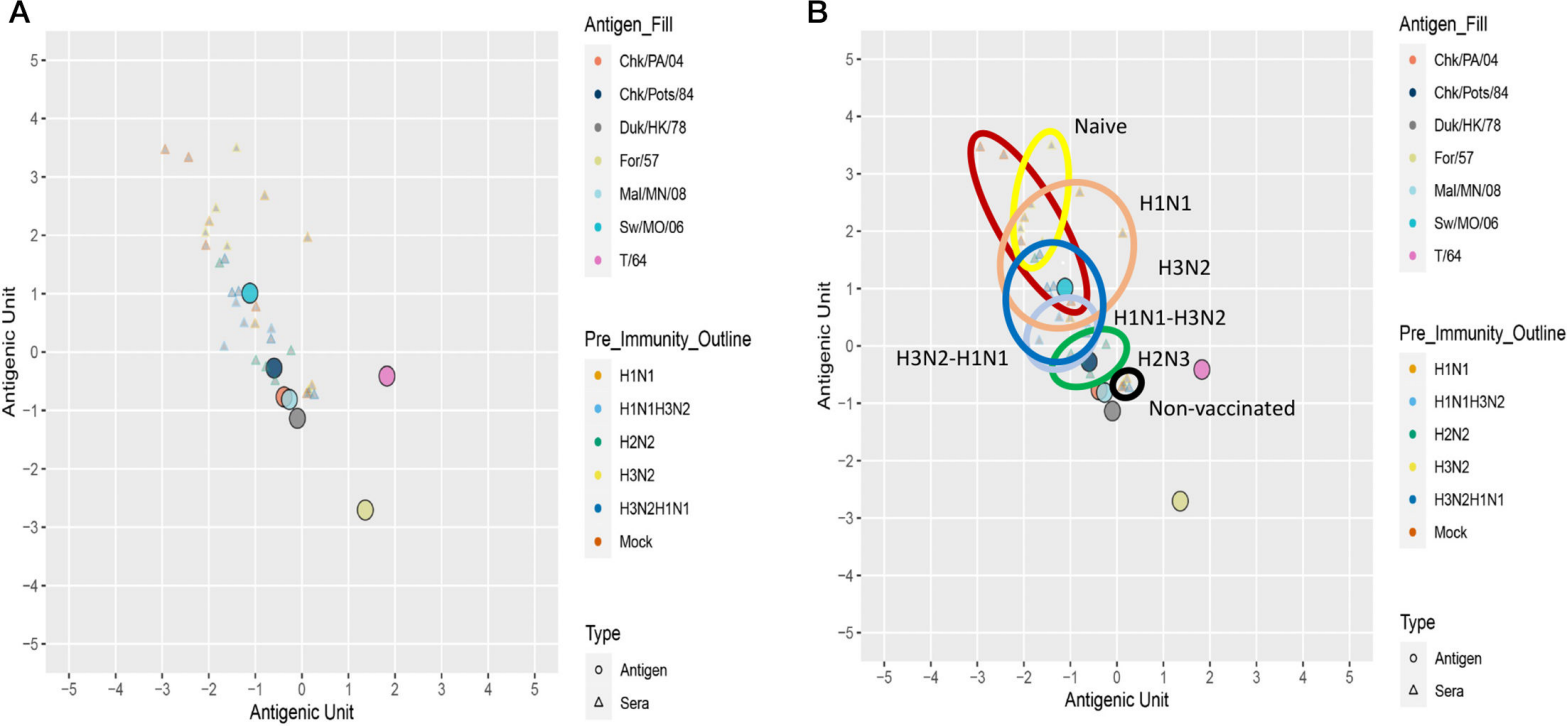
Ferret neutralization antigenic cartography maps after boost vaccination. (**A**) Antigenic maps were generated for each ferret preimmune group in HAI assays. Antigens are represented as circles, and sera from the ferrets are represented by triangles. (**B**) The sera from each influenza preimmune group are grouped within each colored circle for clarity.

The antigenic cartography for the ferret neutralization data showed that the Chk/Pots/84, Chk/PA/04, Duk/HK/78, and Mal/MN/08 antigens clustered together near the center of the map, with the Sw/MO/06, For/57, and T/64 antigens all separated from each other (Fig. 7). The sera from the various preimmune ferret groups had significant overlap, with most sera clustering near most of the antigens at the center of the map. The sera were closest to the Sw/MO/06 antigen, while being furthest from the For/57 and T/64 antigens (Fig. 7). The For/57 virus did not have a mutation in any site identified by Linster et al. (5). The For/57 virus did not have a mutation in any site identified by Linster et al. (5), which indicates that are likely other amino acid sites responsible for the antigenic variation.

### Antigenic variations in human subjects

Sera from human subjects were evaluated for responses to H2Nx influenza viruses. Serum samples were obtained with informed, written consent in 2016 from participants living in the Athens, GA, USA, area. Detailed information about the human study has been previously published, and the IRB information is below (14). Serum was obtained from each participant prior to receiving the seasonal influenza vaccination (split-inactivated, tetravalent vaccine, Sanofi-Pasteur, Swiftwater, PA, USA). We used this pre-vaccination serum to evaluate H2 influenza virus immunity. Study participants were divided into four age groups with birth years as follows: 1934–1951, 1952–1966, 1967–1981, and 1982–1996, noting that those born between 1934 and 1966 were likely exposed to H2 strains, while those born after 1967 were likely not exposed. The oldest two age groups (1934–1951 and 1952–1966) had average geometric mean HAI titers that were significantly higher against 13 of the 16 H2 VLPs than serum from participants in the youngest two age groups (1967–1981 and 1982–1996) (*P*<0.05) (Table 4). The oldest two age groups (1934–1951 and 1952–1966) had average geometric mean HAI titers that were statistically higher (*P* < 0.05) than HAI titers in the 1967–1981 age group to the Duk/HK/78 virus, but there was no statistically significant difference in HAI titers against the 1982–1996 age group (Table 4). There were no statistically significant differences in HAI titers between participants in any of the age groups against either the Musk/Rus/14 or the Chk/Pots/84 VLPs (Table 4).

**TABLE 4:** Geometric mean human HAI titers^*a*^

Age group	J/57	T/64	Duk/HK/78	Mosc/65	Guiy/57	Mal/NL/01	Musk/RU/14	Chk/Pots/84	Duk/Cam/13	Mal/MD/01	Av/Mass/90	Mal/WI/08	Qu/RI/16	Sw/MO/06	Tur/CA/08	GWT/OH/86
1934–1951	6.45 (±1.22)	3.50 (±1.36)	3.43 (±1.45)	5.78 (±1.34)	4.60 (±1.29)	5.12 (±1.31)	2.87 (±1.31)	3.48 (±1.35)	4.69 (±1.42)	6.03 (±1.41)	3.10 (±1.33)	5.58 (±1.28)	5.95 (±1.30)	6.33 (±1.19)	6.90 (±1.16)	6.94 (±1.22)
1952–1966	4.33 (±1.67)	3.79 (±1.47)	3.05 (±1.52)	4.39 (±1.59)	3.65 (±1.57)	4.00 (±1.58)	3.18 (±1.48)	3.34 (±1.45)	4.04 (±1.54)	4.68 (±1.62)	2.82 (±1.36)	3.87 (±1.63)	4.60 (±1.61)	4.56 (±1.61)	4.80 (±1.61)	4.68 (±1.61)
1967–1981	2.39 (±1.11)	2.35 (±1.06)	2.35 (±1.06)	2.35 (±1.06)	2.35 (±1.06)	2.32 (±1.00)	2.32 (±1.00)	2.32 (±1.00)	2.42 (±1.17)	2.39 (±1.18)	2.45 (±1.17)	2.32 (±1.00)	2.32 (±1.00)	2.32 (±1.00)	2.32 (±1.00)	2.32 (±1.00)
1982–1996	2.39 (±1.11)	2.35 (±1.06)	2.36 (±1.06)	2.35 (±1.06)	2.35 (±1.06)	2.32 (±1.00)	2.32 (±1.00)	2.32 (±1.00)	2.42 (±1.17)	2.39 (±1.18)	2.45 (±1.17)	2.32 (±1.00)	2.32 (±1.00)	2.32 (±1.00)	2.32 (±1.00)	2.32 (±1.00)

^*a*^ The geometric mean HAI titers (log2) for individuals in our human cohort against 12 H2 VLPs are shown. Standard deviations for each data point are shown in parentheses. Sera were obtained from individuals with informed consent and were taken prior to influenza vaccination or infection within the previous 6 months. The first column has the birth year of individuals within the group, while remaining columns show the geometric mean HAI titer for each of the 12 H2 VLPs in our panel.

**Fig 8:**
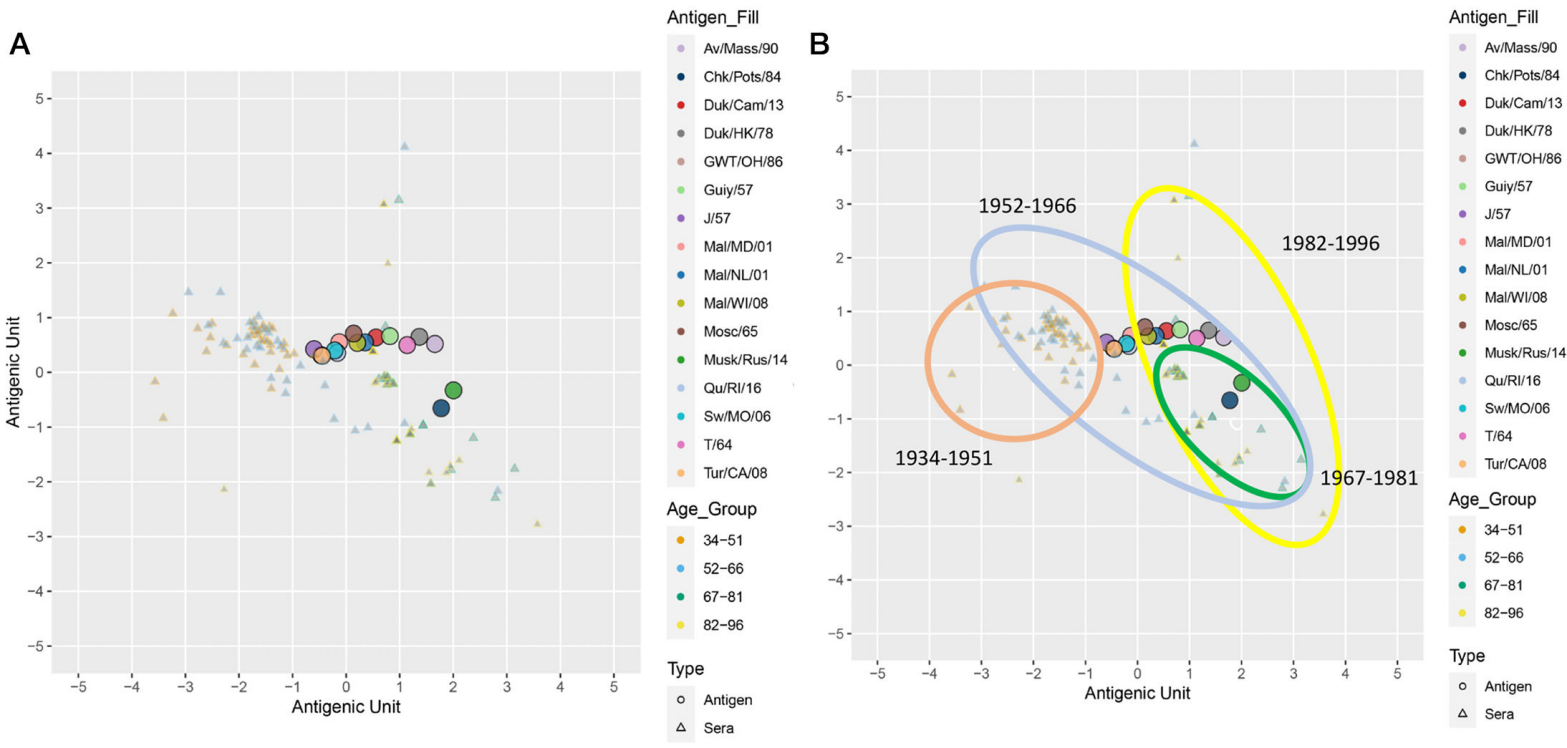
Human HAI antigenic cartography maps. (**A**) Antigenic maps were generated using a cohort of sera from human subjects in HAI assays. Antigens are represented as circles, and sera from the humans are represented by triangles and divided by birth year ranges. (**B**) The sera from human subjects are divided into groups by years of birth and are grouped within each colored circle for clarity.

The antigenic cartography for the HAI titers indicated that most of the antigens clustered together near the center of the graph, with the Musk/Rus/14 and Chk/Pots/84 antigens being slightly separated from the other antigens (Fig. 8). The sera of the 1934–1951 participants clustered relatively close to each other and several antigenic units away from the 1967–1981 and 1982–1996 birth years age groups (Fig. 8). The sera from the 1967–1981 and 1982–1966 groups overlapped but had high variability between participants (Fig. 8). The serum collected from participants in the 1952–1966 group had high variation and was several antigenic units apart (Fig. 8). This likely reflects that these participants were born both before and after H2N2 entered the human population (Fig. 8). The participants born in the mid-1960s also may not have been exposed to H2N2 influenza viruses before these viruses stopped circulating in the human population.

To differentiate the sera from individuals born between 1952 and 1966, we generated antigenic maps of just these individuals (Fig. S7). Most of the HA antigens clustered at the center of the map similarly to the map of the entire population (Fig. S7). The Chk/Pots/84 and Musk/Rus/14 antigens are divergent from the other antigens. The T/64, Av/Mass/90, and Duk/HK/78 HA antigens also separated from the other HA antigens (Fig. S7). Most of the sera clustered near the center of the map, with a few serum samples being highly divergent (>2 antigenic units) (Fig. S7).

For neutralization titers, participants in the two oldest age groups had high neutralization titers (>1:400) to Sw/MO/06 and intermediate responses against the other H2 strains, potentially reflecting H1 immune breadth against H2 strains and the circulation of H2N2 strains after 1956 (Table 5). The two oldest age groups had the highest geometric mean neutralization titers (>1:100) against the panel of viruses (Table 5). The participants in the 1952–1966 were the only group to have a geometric mean neut titer of ≥1:100 for more than one virus (Table 5). On average, participants in the 1967–1981 age group had reduced/low-level H2 titers across the panel. Sw/MO/06 was also the only virus that the 1967–1981 age group had which demonstrated neutralization titers >1:25 (Table 5). As expected, participants in the youngest age group had low neutralization titers (<1:10) against all viruses in the panel (Table 5).

**TABLE 5:** Geometric mean of human neutralization titers^*a*^

Age groups	Chk/Pots/84	Chk/PA/04	Form/57	T/64	Duk/HK/78	Sw/MO/06	Mal/MN/08
1934–1951	3.98 (±1.40)	3.72 (±1.32)	4.99 (±1.52)	4.49 (±1.29)	4.15 (±1.39)	8.66 (±1.10)	4.28 (±1.41)
1952–1966	4.48 (±1.58)	4.3 (±1.42)	3.60 (±1.54)	5.27 (±1.35)	4.54 (±1.57)	8.58 (±1.11)	4.57 (±1.59)
1967–1981	3.09 (±1.29)	3.46 (±1.23)	3.09 (±1.29)	3.93 (±1.27)	2.32 (±1.00)	4.31 (±2.40)	3.28 (±1.63)
1982–1996	2.32 (±1.00)	2.32 (±1.00)	2.32 (±1.00)	2.85 (±1.27)	2.60 (±1.18)	2.39 (±1.06)	2.6 (±1.18)

^*a*^ The geometric mean neutralization titers (log2) for individuals in our human cohort against seven H2 influenza viruses is shown. Standard deviations for each data point are shown in parentheses. Sera were obtained from individuals with informed consent and were taken prior to influenza vaccination or infection within the previous 6 months. Column 1 has the birth year of individuals within the group, while remaining columns show the geometric mean neut titer for each of the seven H2 influenza viruses in our panel.

**Fig 9:**
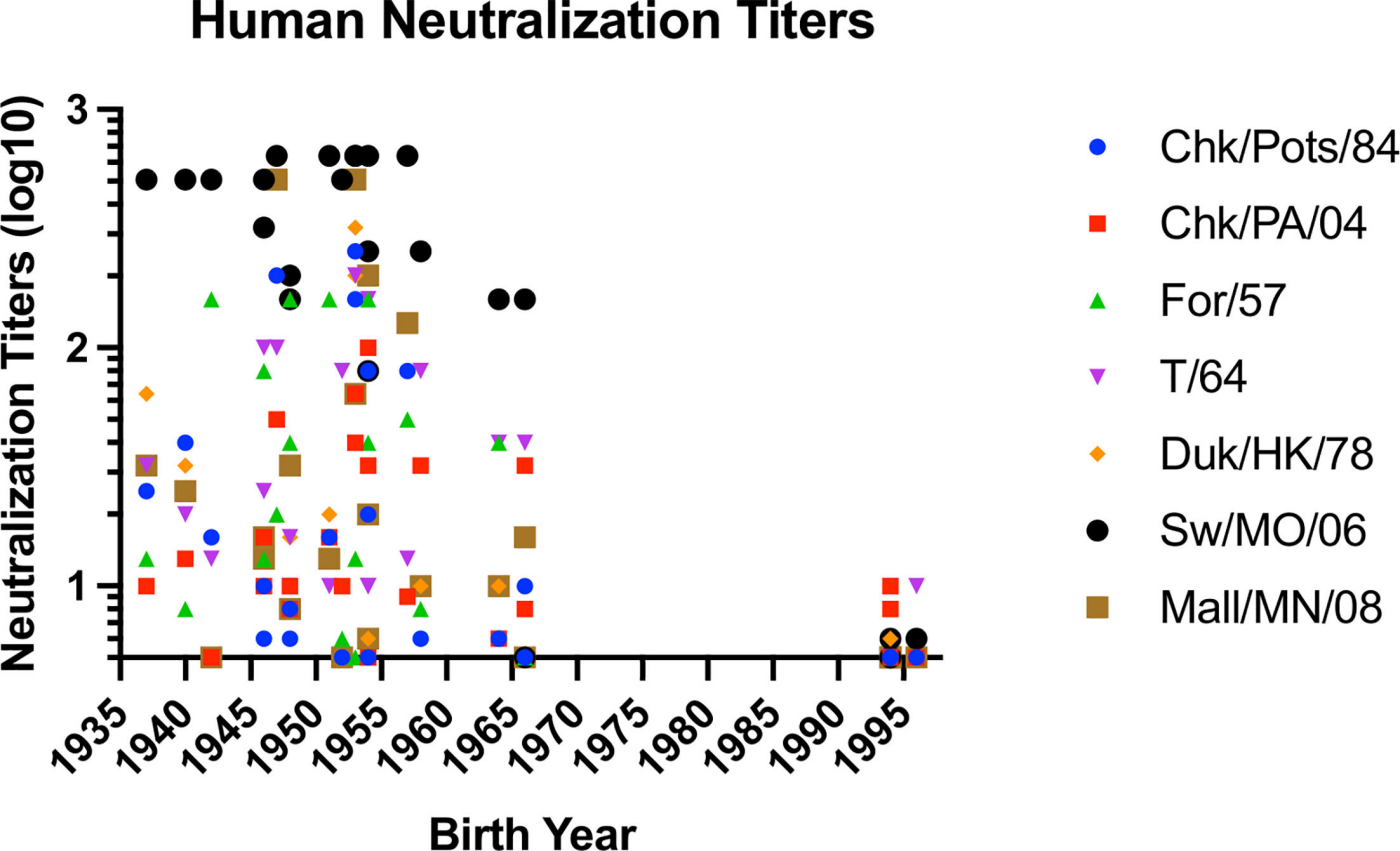
Neutralization of H2Nx influenza viruses with human sera by birth year. The neutralization titers for individuals in our human cohort against seven H2Nx influenza viruses are separated by each individual’s birth year. Sera were obtained from individuals with informed consent and were taken prior to influenza vaccination or infection within the previous 6 months. The *x*-axis shows the birth year of each individual, with the *y*-axis showing the neutralization titer (log10). The older individuals have higher and more robust neutralization titers than the individuals born in the 1990s.

We also generated antigenic cartography maps and scatter plots to visualize the human H2 influenza virus neutralization data (Fig. S8, Fig. 9). For the scatter plot, individuals born in the 1960s or before had higher neutralization titers for each of the seven H2 influenza viruses compared to individuals born in the 1990s (Fig. 9). The individuals born in the 1990s had neutralization titers at or near the limit of detection for each of the seven viruses in our panel (Fig. 9). The antigenic cartography for the neutralization assays reveals that all of the antigens except Sw/MO/06 and For/57 cluster together (Fig. S8). Sw/MO/06 is at the center of the map, while For/57 is several antigenic distance units from all of the other HA antigens as well as all of the sera (Fig. S8).

## DISCUSSION

Using antigenic cartography to map the antigenic distances between H2 influenza virus strains, we re-analyzed both HAI and neut data in both ferrets and in humans from previous studies to evaluate the impact of variable single and multiple H1 and H3 infection pre-exposure histories on H2 vaccine performance (13, 14). Under controlled experimental conditions, these studies collectively reveal the stark differences in H2 influenza antigenic recognition, depending upon exposure history. In particular, a primary H1N1 infection or H1N1 + H3N2 infection elicits imprinted responses that effectively promote robust immune titers following H2 vaccination, while H3N2 primary infections (regardless of H1N1 secondary infection) attenuate H2 vaccine responses. Obviously, these conclusions are based on single exposures to these strains and may underrepresent the impact of multiple, sequential infections on H2 vaccine performance.

However, the H1N1-imprinted ferrets likely responded better than the H3N2-imprinted ferrets to our H2 HA vaccines because both H1 and H2 influenza HAs are group 1 HA proteins, while H3 is a group 2 HA protein. Previous studies have shown that immune imprinting with a group 1 HA is correlated with lower infection rates with heterologous group 1 HA-containing influenza viruses, while immune imprinting with a group 2 HA is correlated with lower infection rates with heterologous group 2 HA-containing influenza viruses (11, 12). Additionally, influenza virus group 1 and group 2 HA stem vaccines have shown some effectiveness in protecting against multiple heterologous HAs within each group (19–21). Taken together, these findings suggest that the memory B cells present after H1N1 virus infections were able to be activated in the presence of the closely related H2 HA antigens.

Ferrets, pre-exposed to either H1N1 or H3N2 alone or after sequential infection with both viruses, elicited little, if any, cross-neutralization titers directed against H2N2 influenza viruses isolated over a 50 year period. After rH2 vaccination, H2N3-preimmune ferrets had neutralization titers that increased following vaccination with homologous H2 HA antigens against all H2 subvariants in the panel, although some strains were more resistant to neutralization than others. In contrast, ferrets pre-exposed to H3N2 influenza viruses either alone or prior to H1N1 influenza virus infection had reduced titers to all of the H2 vaccines compared with the other preimmune groups. Importantly, these differential immune responses can be modulated in part, using the Z1 COBRA immunogen, which elicited superior H2 responses compared with the other H2 HA antigens tested.

One likely interpretation of these data is that H2 HA vaccines may be less effective in preimmune adult populations, depending on exposure history and the sequence of H1N1 and H3N2 infection/vaccine history, as well as host genetic variation. The data also suggest that naïve infants will respond differently than adult populations after prime vaccination or infection due to the absence of memory B cells. Additional studies are needed to evaluate the impact that vaccination following initial influenza exposure has on future influenza vaccinations, as opposed to infections. Moreover, these studies reveal that factors such as preimmune status, strains used in the viral panel, and host genetic background can obfuscate the results of vaccine studies and should be carefully considered in future clinical studies.

H2 HA vaccine boosters clearly enhanced global H2 neutralization responses across the panel, regardless of vaccine group, with interesting caveats. Overall, the Z1 COBRA HA vaccine was clearly superior to all other formulations as measured by HAI and neutralization titers. Still, one H2 HA (Musk/RUS/14) was clearly resistant to neutralization, suggesting an important role for strain-specific variation in altering neutralization performance and potentially leading to breakthrough infections. After the second vaccination, both the H3N2 and H3N2-H1N1 preimmune ferrets had higher H2 HAI titers across the panel, although overall, these were slightly lower than the titers in H2 preimmune, H2-vaccinated ferrets. The stark differences between the first and second vaccinations in the HAI assay suggest the need for multiple vaccinations in the event of a future H2Nx influenza pandemic. This would be similar to the multiple vaccinations required to generate robust antibodies against the SARS-CoV-2 virus, since people had no pre-existing immunity to this virus that causes COVID-19. Most people under the age of 60 do not have pre-existing neutralizing antibodies against H2Nx influenza viruses (14).

While our studies focused on the immunity to the HA protein of influenza, NA immunity has recently emerged as another important area of influenza immunity (22–25). Recent studies have shown that NA-specific antibodies generated from both H2N2 and H3N2 infections elicit antibodies that cross-react with avian origin H9N2 viruses. Future studies of both our ferret and human sera could elicit further insights into NA preimmunity and imprinting, as we have shown with HA immunity. However, we do not believe that NA immunity affected either our HAI or neutralization results. This is due to both the mock-vaccinated, H3N2 preimmune ferrets and the humans born after 1970 having both HAI and neutralization titers at or below the limit of detection against H2N2 influenza VLPs or viruses. While NA-directed antibodies can theoretically affect HAI titers by causing steric hindrance, other studies, including ours, show that the presence of NA antibodies does not always affect HAI titers (25). Other studies have demonstrated that H3N2 preimmune ferrets are protected from H2N2 viral challenge. Future studies should examine the effects of both HA and NA immunity both in singluarity and in combination.

This study also utilized data generated by Linster et al. (5), who identified important antigenic sites in HA by rescuing human-derived H2N2 influenza viruses with single amino acid mutations. These mutant HA sequences were compared on the antigenic maps generated using HAI and neutralization titers. Some mutations found by Linster et al. likely explain the divergence of antigens, such as Musk/Rus/14 and T/64 (5). The data from this study suggest that additional pivotal antigenic sites at positions 140 and 151 may contribute to the divergence of Musk/Rus/14, T/64, and Duk/HK/78 either singly or in combination, although there are numerous sites where Duk/HK/78 encodes unique amino acids. The high number of unique mutations in Duk/HK/78 would require extensive additional studies to ascertain which sites are causing antigenic divergence. These sites may not have been identified previously because the human-derived H2N2 viruses did not contain mutations at either of these amino acid locations. We attempted to elevate the importance of both antigenic sites, but we were unable to show statistically significant HAI changes for either. Additional studies are needed to investigate the antigenic importance of each mutation in Musk/Rus/14 HA, both individually or in combination.

Our studies also demonstrate the need for continuous evaluation of the biosafety risk of viruses. Until 2005, H2N2 human viruses were classified as BSL2 viruses (26). All human strains of H2N2 are now classified as BSL3 viruses. However, avian strains of H2Nx viruses were still classified as BSL2 viruses. Indeed, the data obtained for our studies were collected using BSL2 safety conditions. Our studies showed a lack of human immunity to the majority of the H2Nx influenza viruses. This lack of immunity demonstrates the need for constant evaluation and reevaluation of biosafety risks that viruses pose and the importance of maintaining appropriate biosafety under changing human immunity and virus evolution.

## MATERIALS AND METHODS

### COBRA HA antigen design

The design of each COBRA vaccine has been previously published in detail (18). Briefly, amino acid sequences from WT H2Nx strains were downloaded from the Global Initiative on Sharing All Influenza Data. The original amino acid sequences were grouped together to create primary consensus sequences. This layering consensus method was continued until final consensus sequences were obtained. These COBRA amino acid sequences were then reverse translated into nucleotide sequences and codon-optimized for mammalian expression to create VLPs.

In response to the intensive task of applying the COBRA method within Geneious Prime, a program was designed leveraging the Geneious Prime Application Programming Interface and Java to streamline the process by automating several key steps. First, the program automates the alignment of amino acid sequences, ensuring that despite sequence diversity, all amino acids at corresponding positions are oriented. Next, specific regions of proteins, typically variable ones, are automatically selected, truncated, and followed by a second round of alignment. Subsequently, the program groups the realigned sequences based on a predetermined similarity threshold, effectively clustering sequences that share resemblance to a reference sequence. Within each group, it generates a consensus sequence representing the most common amino acid at each position across the sequences. The various groupings of consensus sequences establish a primary layer, which serves as the foundation for further refinement.

Following the creation of the primary layer, the program iteratively groups consensus sequences from the previous layer into a new layer, continuing this process until convergence to a single sequence suitable for vaccine development is obtained. Through automation, the program has significantly expedited the implementation of the COBRA method within Geneious Prime, streamlining the process of identifying potential vaccine candidates from diverse amino acid sequences while minimizing human error.

### Ferret vaccinations

Fitch ferrets (*Mustela putorius furo*, spayed, female, 6 to 12 months of age) were purchased certified influenza-free and de-scented from Triple F Farms (Sayre, PA, USA). Ferrets were pair housed in stainless steel cages (Shor-Line, Kansas City, KS) containing Sani-Chips laboratory animal bedding (P. J. Murphy Forest Products, Montville, NJ). Ferrets were provided with Teklad Global Ferret Diet (Harlan Teklad, Madison, WI) and fresh water *ad libitum*. Ferrets (*n* = 20) were infected with H1N1, H3N2 seasonal influenza viruses, or H2N2 avian influenza viruses in different orders before vaccination. The H1N1 influenza viruses used to establish preimmunity are Singapore/6/1986 (Sing/86) and California/07/2009 (Cal/09), the H3N2 influenza viruses used to establish preimmunity are Sichuan/2/1987 (Sich/87) and Panama/2007/1999 (Pan/99), and the H2N2 avian influenza viruses used to establish preimmunity are Chk/PA/04 and Qu/RI/16, all at an infectious dose of 1e+6 PFU in 1 mL intranasally. For the ferrets with multiple preimmune infections, ferrets were left for 60 days between each infection and before the first vaccination.

After the establishment of preimmunity by viral infection, 60 days elapsed before ferrets were vaccinated with rHA twice, with 4 weeks between vaccinations. The ferrets were vaccinated with a 1:1 ratio (500 μL total volume) of rHA diluted with PBS (15.0 μg rHA/ferret), and the emulsified oil-water adjuvant Addavax (InvivoGen, San Diego, CA, USA). The mock-vaccinated groups received only PBS and Addavax adjuvant at a 1:1 ratio (500 μL total volume) with no rHA. Each vaccination was given intramuscularly. Before vaccination and 2 weeks after each vaccination, ferrets were bled, and serum was isolated from each sample. The blood was harvested from all anesthetized ferrets via the anterior vena cava on days 0, 14, and 42. Blood samples were incubated at room temperature for 1 h prior to centrifugation at 6,000 rpm for 10 min. The separated serum was removed and frozen at −20°C.

### Mouse vaccinations

BALB/c mice (females, 6 to 8 weeks old) were purchased from The Jackson Laboratory (Bar Harbor, ME, USA) (Stock #000671). The mice were housed in microisolator units and were given both water and food *ad libitum*. The mice were vaccinated with a 1:1 ratio (50 μL total volume) of rHA diluted with PBS (3.0 μg rHA/mouse) and the emulsified oil-water adjuvant, AddaVax (InvivoGen, San Diego, CA, USA). The mice were boosted with the same vaccine formulation, with the same dosage, at 4 weeks post-initial vaccination. Blood samples were obtained from the mice via cheek bleeds 14 to 18 days following the second vaccination. Blood samples were collected in 1.5 mL microcentrifuge tubes. The blood samples were incubated at room temperature for 1 h and then centrifuged at 6,000 rpm for 10 min. Serum samples were transferred to new 1.5 mL microcentrifuge tubes and stored at −20°C.

### Human participants and vaccinations

Participants ranging between the ages of 19 and 82 years old consented to the study and were enrolled in Athens, GA, USA. Various factors were utilized to determine the participants’ eligibility. Those who had not yet received the seasonal influenza virus vaccine at the time of enrollment, the beginning of September 2016, were still included in the study (University of Georgia, IRB# STUDY00003773). Influenza strains included in the vaccine were based upon the WHO recommendations for the Northern Hemisphere: (A/California/7/2009-H1N1), (A/Hong Kong/4801/2014 H3N2), (B/Phuket/3073/2013-Yamagata-lineage), (B/Brisbane/60/2008-Victoria-lineage). Participants received vaccinations from September 2016 to December 2016. Participants were vaccinated with the standard dose (15 μg/antigen) split-virion (IIV) version of licensed Fluzone (Sanofi Pasteur) influenza virus vaccine. One hundred forty-eight participants were enrolled in the study. Approximately 80 mL of blood was collected from each participant before vaccination (D0), 7 days post-infection (D7), and 21 days post-infection (D21). Sera and peripheral blood mononuclear cells (PBMCs) were isolated from the blood samples. Sera were collected in Vacutainer serum separation tubes (BD Biosciences) and processed within 48 h, aliquoted, and stored at −20 °C. PBMCs were collected in Vacutainer cell preparation tubes (BD Biosciences) at D0, D7, and D21. PBMC samples suspended in DMSO and FBS and were stored in liquid nitrogen.

### Viruses, rHA antigens, and VLPs

The following were obtained from either the United States Department of Agriculture’s Diagnostic Virology Laboratory in Ames, IA; BEI Resources; or provided by the laboratory of Dr. S. Mark Tompkins in Athens, GA: A/Chicken/Potsdam/4705/1984 (Chk/Pots/84, H2N2), A/Chicken/PA/298101-4/2004 (Chk/PA/04, H2N2), A/Duck/Hong Kong/273/1978 (Duk/HK/78, H2N2), A/Mallard/Minnesota/AI08-3437/2008 (Mal/MN/08, H2N3), A/Swine/Missouri/4296424/2006 (Sw/MO/06, H2N3), A/Formosa/313/1957 (For/57, H2N2), A/Japan/305/1957 (J/57, H2N2), and A/Taiwan/1/1964 (Tw/64, H2N2). The following H1N1 influenza viruses used in the study were provided by either the Centers for Disease Control and Prevention (CDC) or Virapur LLC: A/Singapore/6/1986 (Sing/86), A/California/07/2009 (Cal/09; pandemic). Passaged using embryonated chicken eggs, each virus was harvested from the eggs and aliquoted into tubes, which were stored at −80°C. Each virus was titrated using a standard influenza plaque assay.

rHA proteins were produced using the pcDNA 3.1+ plasmid. Each HA gene was truncated by removing the transmembrane (TM) domain and the cytoplasmic tail at the 3′ end of the gene. The TM domain was determined using the TMHMM Server v.2.0 website: http://www.cbs.dtu.dk/services/TMHMM/. The HA gene was truncated at the first amino acid prior to the TM domain. A fold-on domain from T4 bacteriophage, an Avitag, and a 6× histidine tag totaling 477 nucleotides were added to the 3′ end of the HA gene. The pcDNA 3.1+ vectors were then transfected individually into human endothelial kidney 293T (HEK293T) suspension cells using ExpiFectamine 293 transfection reagent following manufacturer’s specifications (Thermo Fisher Scientific). The supernatants were then harvested from the transfected HEK293T cells. rHAs were purified from the supernatant using a nickel-agarose column. The rHAs were then eluted from the column using imidazole. After elution, proteins were quantified using bicinchoninic acid (BCA) assay and stored at −80°C.

For the VLP production, HEK293T cells (1 × 10^6^) were transiently transfected for the creation of mammalian VLPs. DNA of each of the three pcDNA 3.1+ mammalian expression vectors expressing the influenza neuraminidase (A/South Carolina/1/1918; H1N1), the HIV p55 Gag sequence, and one of the various H2 wild-type HA proteins was added in a 1:2:1 ratio with a final DNA concentration of 1 μg. Following 72 h of incubation at 37°C, supernatants from transiently transfected cells were collected, centrifuged to remove cellular debris, and filtered through a 0.22 μm pore membrane. VLPs were purified and sedimented by ultracentrifugation on a 20% glycerol cushion at 23,500 × *g* for 4 h at 4°C. VLPs were resuspended in PBS, and total protein concentration was determined with the Micro BCA Protein Assay Reagent kit (Pierce Biotechnology, Rockford, IL, USA). Hemagglutination activity of each preparation of VLP was determined by serially diluting volumes of VLPs and adding equal volume of 0.8% turkey red blood cells (RBCs) (Lampire Biologicals, Pipersville, PA, USA) suspended in PBS to a V-bottom 96-well plate with a 30 min incubation at room temperature (RT). Prepared RBCs were stored at 4°C and used within 72 h. The highest dilution of VLP with full agglutination of RBCs was considered the endpoint HA titer. The HA sequences used for VLPs were Mal/NL/01, Chk/Pots/84, Muskrat/Russia/63/2014 (Musk/Rus/14), Duck/Cambodia/419W12M3/2013 (Duk/Cam/13), J/57, Moscow/1019/1965 (Mosc/65), T/64, Duk/HK/78, Mal/WI/08, Sw/MO/06, Quail/Rhode Island/16-018622-1/2016 (Qu/RI/16), Turkey/California/1797/2008 (Tur/CA/08).

### Hemagglutination inhibition assay

HAI assays were used to quantify receptor-binding HA-specific antibodies through measuring the inhibition in the agglutination of turkey RBCs. We utilized VLPs for our HAI assay due to lack of available live viruses. Previous studies have demonstrated that VLPs and live viruses are intertangled in the HAI assay and do not affect assay results (27). Prior to being tested, the sera were treated with receptor-destroying enzyme (RDE) (Denka Seiken Co., Japan) to inactivate nonspecific inhibitors. Three parts RDE were added to one part sera and incubated overnight at 37°C. RDE was subsequently inactivated by incubating the serum-RDE mixture at 56°C for approximately 45 min. After the incubation period, six parts PBS were added to the RDE-treated sera. The RDE-treated sera were twofold serially diluted in V-bottom microtiter plates. An equal volume of each VLP was adjusted to approximately 8 hemagglutination units/25 μL and added to each well of the V-bottom microtiter plates. The plates were covered and incubated at RT for 20 min before adding 50 μL of RBCs, which were allowed to settle for 30 min at RT. The HAI titer was determined by the reciprocal dilution of the last well that contained non-agglutinated RBCs.

### Neutralization assays

All neutralization assays were performed in BLS3 biocontainment facilities at the University of Georgia regardless of the biosafety level of the virus. Neutralization assays were used to identify the presence of virus-specific neutralizing antibodies. Antibodies were diluted in 1/2-log increments with serum-free media and incubated with 100 times TCID50 for 1 h. The antibody-virus mixture was then added to the incomplete (FBS-free) DMEM-washed MDCK cells in the 96-well plate. After 2 h, the MDCK cells were washed with incomplete DMEM. Approximately 200 μL of DMEM with P/S and TPCK was added to each of the 96 wells. The cell monolayers were checked daily for cytopathic effect (CPE). CPE was defined as >10% CPE of cells per well. After 3–4 days, media in each well was removed and the MDCK cells were fixed with 10% buffered formalin. MDCK cells were stained using 1% crystal violet (Sigma).

### Exclusion criteria

Exclusion criteria included documented contraindications to Guillain-Barré syndrome, dementia or Alzheimer’s disease, allergies to eggs or egg products, estimated life expectancy <2 years, medical treatment causing or diagnosis of an immunocompromising condition, or concurrent participation in another influenza vaccine research study. Participants were not monitored for influenza virus infection during the time of their participation. Although influenza virus did not circulate widely in the community during this time, participants were asked during each visit if they had experienced flu-like symptoms, and those who did were excluded from the study.

### Antigenic cartography

The antigenic cartography analyses were carried out in R version 4.1.3 (https://www.R-project.org/) with the antigenic and sera coordinates calculated through the racmacs package (https://github.com/acorg/Racmacs) and the graphic displays done with ggplot2. The samples were grouped by either vaccine, preimmunity, or age. The coordinates of the sera and antigens were calculated with a run of 1,000 optimizations to minimize the difference between the n-dimensional Euclidean distances between points, and the two-dimensional distances on the final map.

### Statistics

Statistical significance for assays was defined as a *P*-value of less than 0.05. The limit of detection for HAIs is 1:10. 1:5 was used for statistical analysis. The HAI titers were transformed to log2 for analysis and graphing for one-way ANOVA analysis. Geometric mean titers were calculated for neutralization assays, but the log10 titers were used for ANOVA analysis. All error bars on the graphs represent standard error of the mean.

## Data Availability

All of the raw data used for this study has been previously published (13, 14, 18). Data files from this study can also be found at https://doi.org/10.17605/OSF.IO/9C8YV.

## SUPPLEMENTAL MATERIAL

Fig. S1 msphere.00022-26-s0001.tif HAI titers of ferrets against viruses used to establish preimmunity.

Fig. S2 msphere.00022-26-s0002.tif Cross-reactive antibody responses to H2 VLPs post-H2N2 virus infection.

Fig. S3 msphere.00022-26-s0003.tif Amino acid diversity in WT and COBRA H2 HA sequences.

Fig. S4 msphere.00022-26-s0004.tif The main effects of vaccines received, preimmunity, and virus tested on the log 2 HAI titer post-boost were determined.

Fig. S5 msphere.00022-26-s0005.tif Amino acid diversity in sites identified by Linster et al.

Fig. S6 msphere.00022-26-s0006.tif Amino acid diversity at potentially significant sites.

Fig. S7 msphere.00022-26-s0007.tif Human HAI antigenic cartography maps for 1952–1966 age group.

Fig. S8 msphere.00022-26-s0008.tif Human neutralization antigenic cartography maps.

ASM does not own the copyrights to Supplemental Material that may be linked to, or accessed through, an article. The authors have granted ASM a non-exclusive, world-wide license to publish the Supplemental Material files. Please contact the corresponding author directly for reuse.
