# Supplementary figures and images for: Antigenic mapping of H2 influenza viruses recognized by ferret and human sera and predicting antigenically significant sites
Source: mSphere. 2026 Jun 10;11(6):e00022-26. doi: 10.1128/msphere.00022-26 (PMC13317192; doi:10.1128/msphere.00022-26)

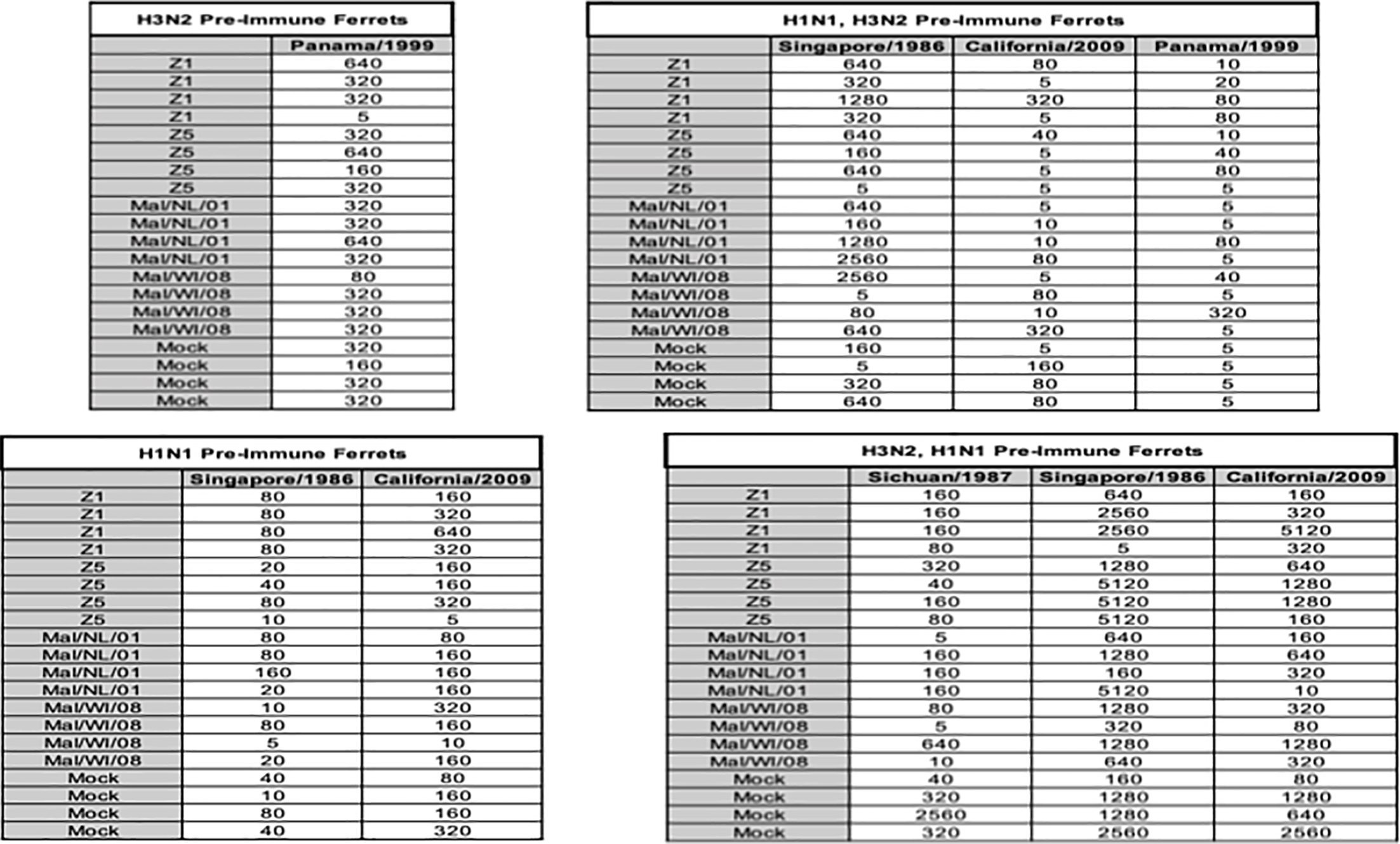

Supplement: Fig. S1 — HAI titers of ferrets against viruses used to establish preimmunity. [file msphere.00022-26-s0001.tif]

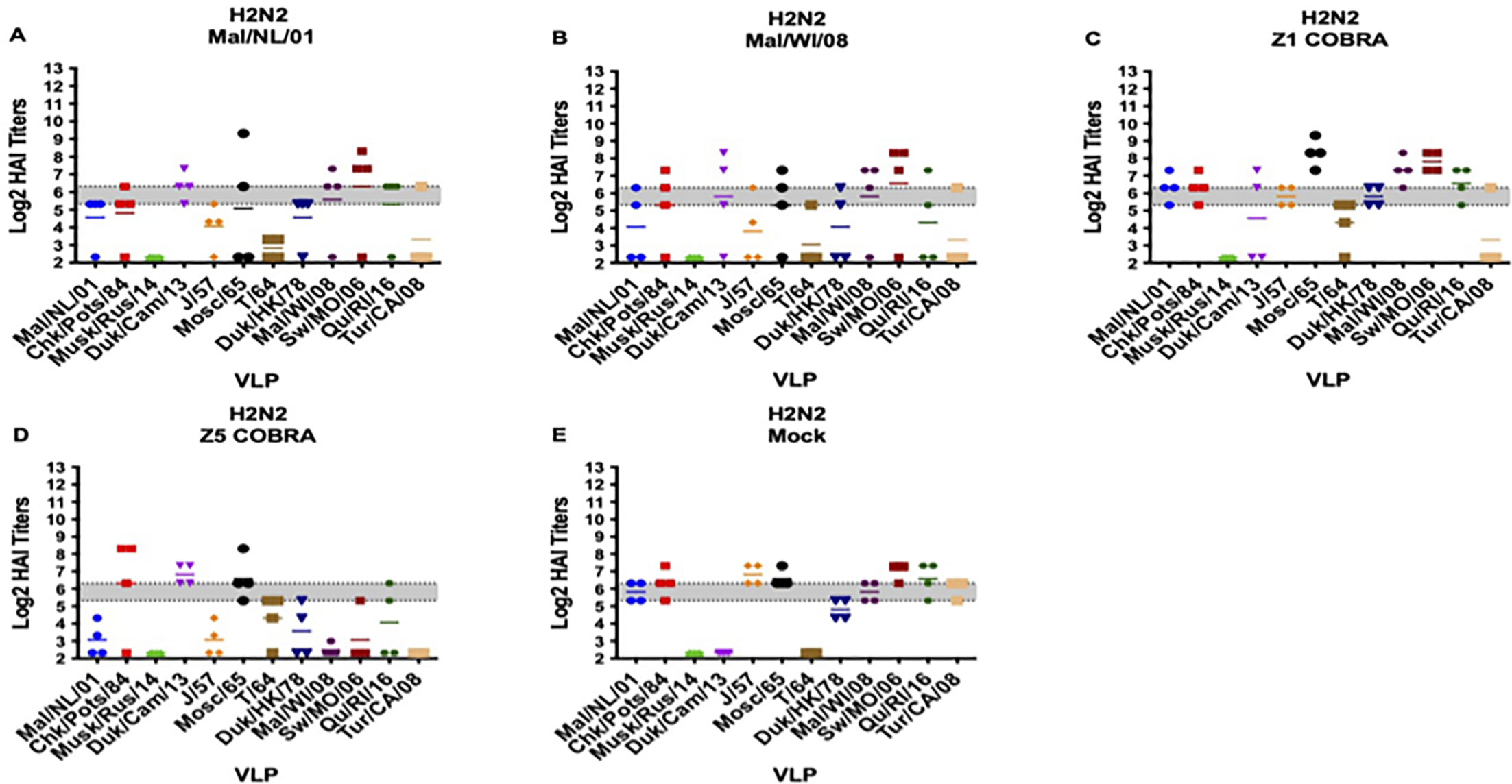

Supplement: Fig. S2 — Cross-reactive antibody responses to H2 VLPs post-H2N2 virus infection. [file msphere.00022-26-s0002.tif]

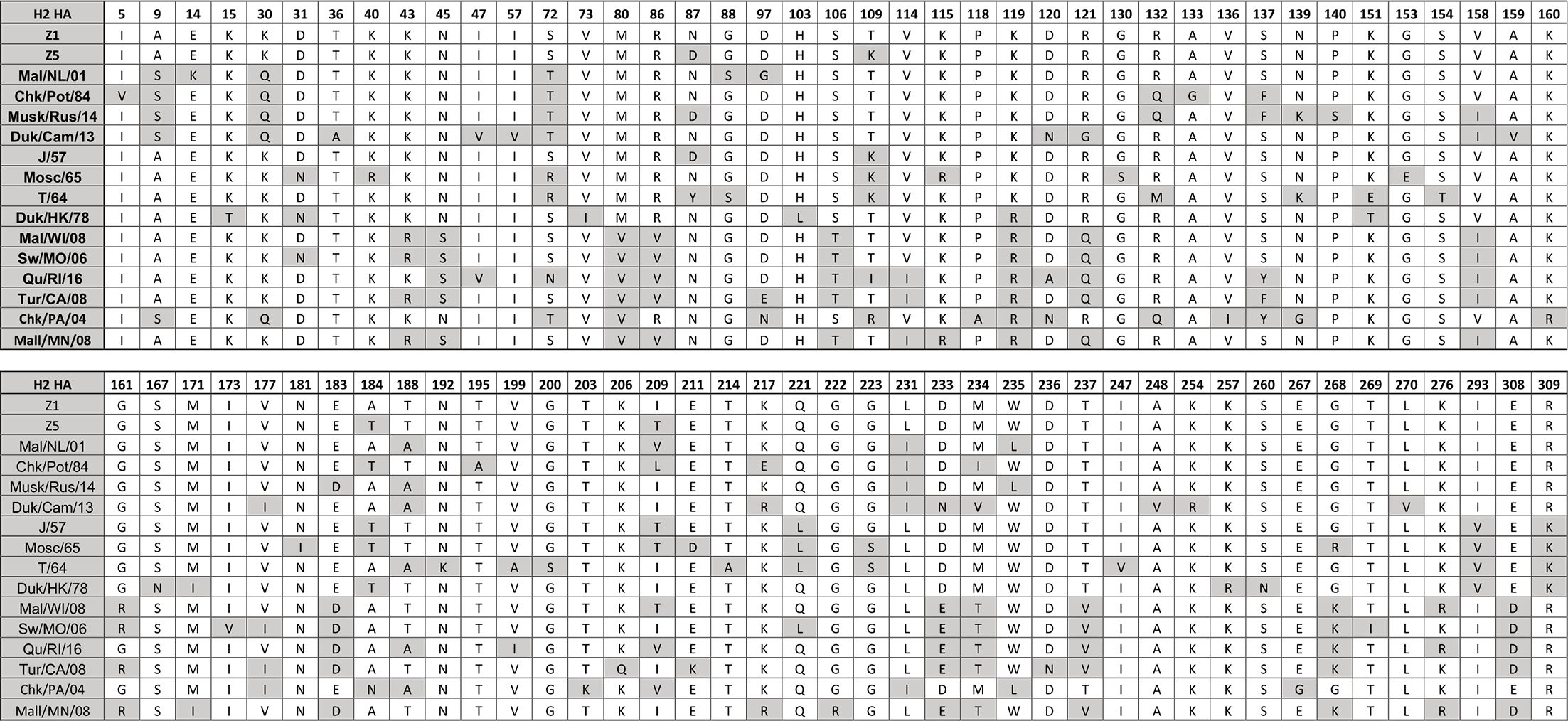

Supplement: Fig. S3 — Amino acid diversity in WT and COBRA H2 HA sequences. [file msphere.00022-26-s0003.tif]

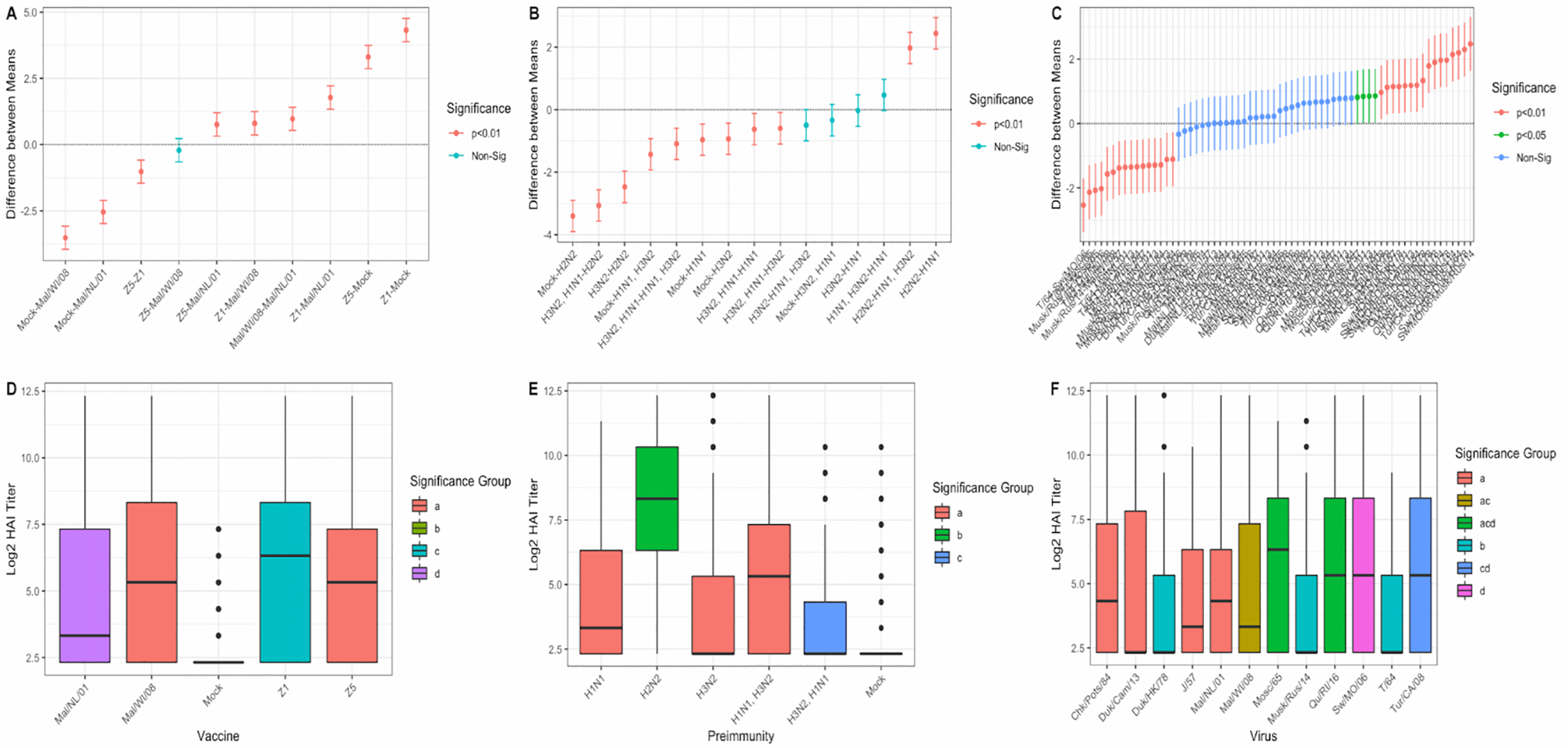

Supplement: Fig. S4 — The main effects of vaccines received, preimmunity, and virus tested on the log 2 HAI titer post-boost were determined. [file msphere.00022-26-s0004.tif]

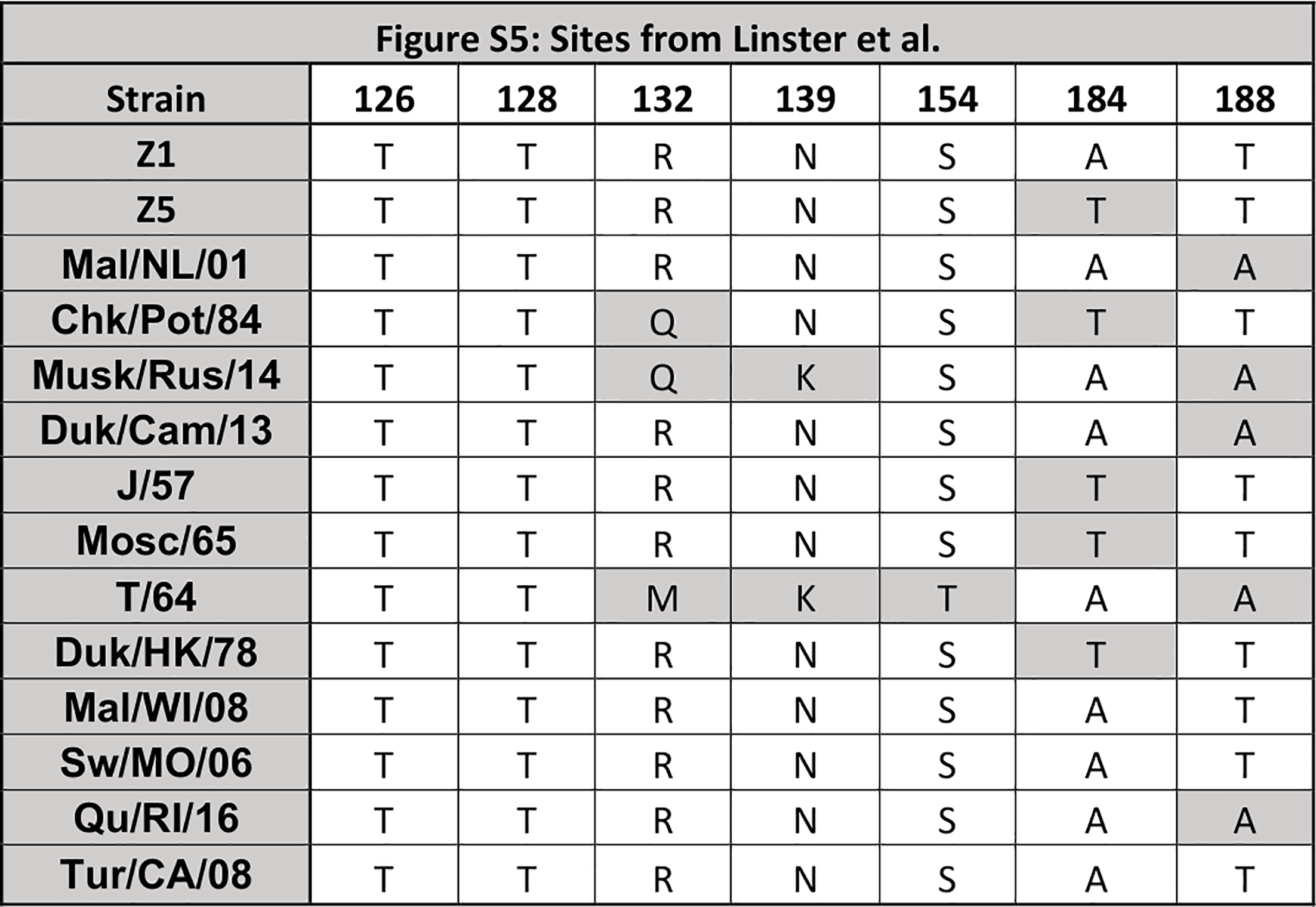

Supplement: Fig. S5 — Amino acid diversity in sites identified by Linster et al. [file msphere.00022-26-s0005.tif]

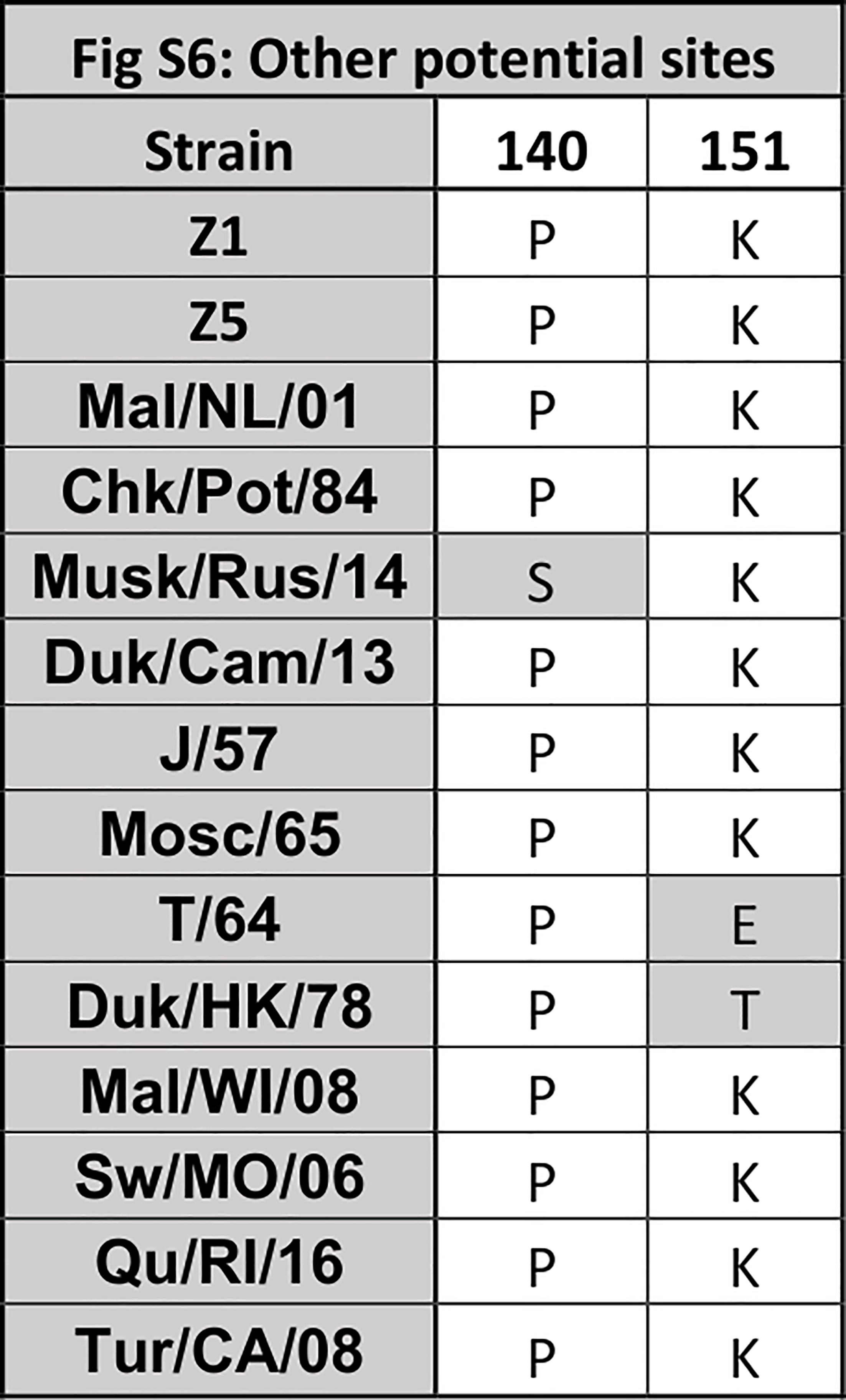

Supplement: Fig. S6 — Amino acid diversity at potentially significant sites. [file msphere.00022-26-s0006.tif]

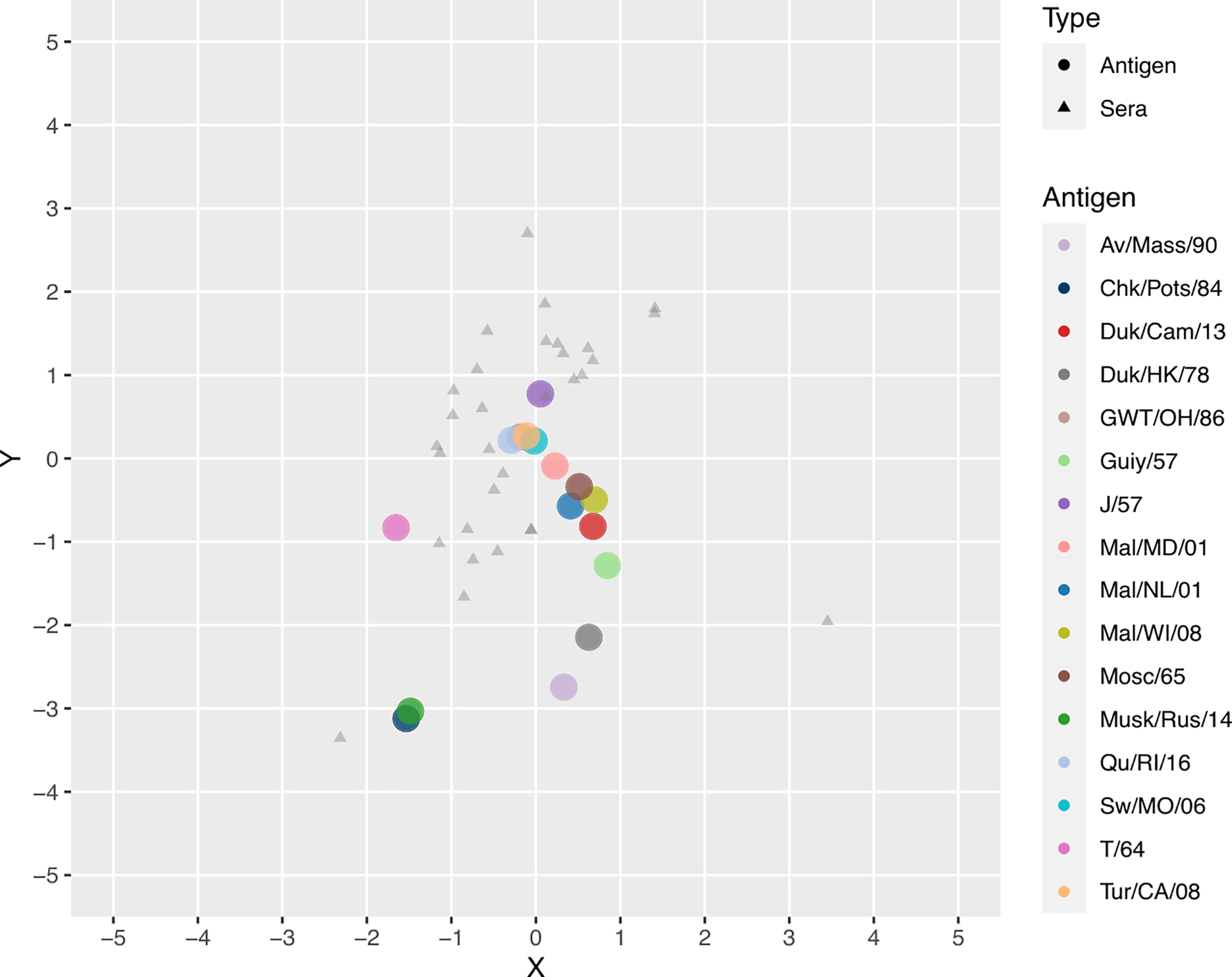

Supplement: Fig. S7 — Human HAI antigenic cartography maps for 1952–1966 age group. [file msphere.00022-26-s0007.tif]

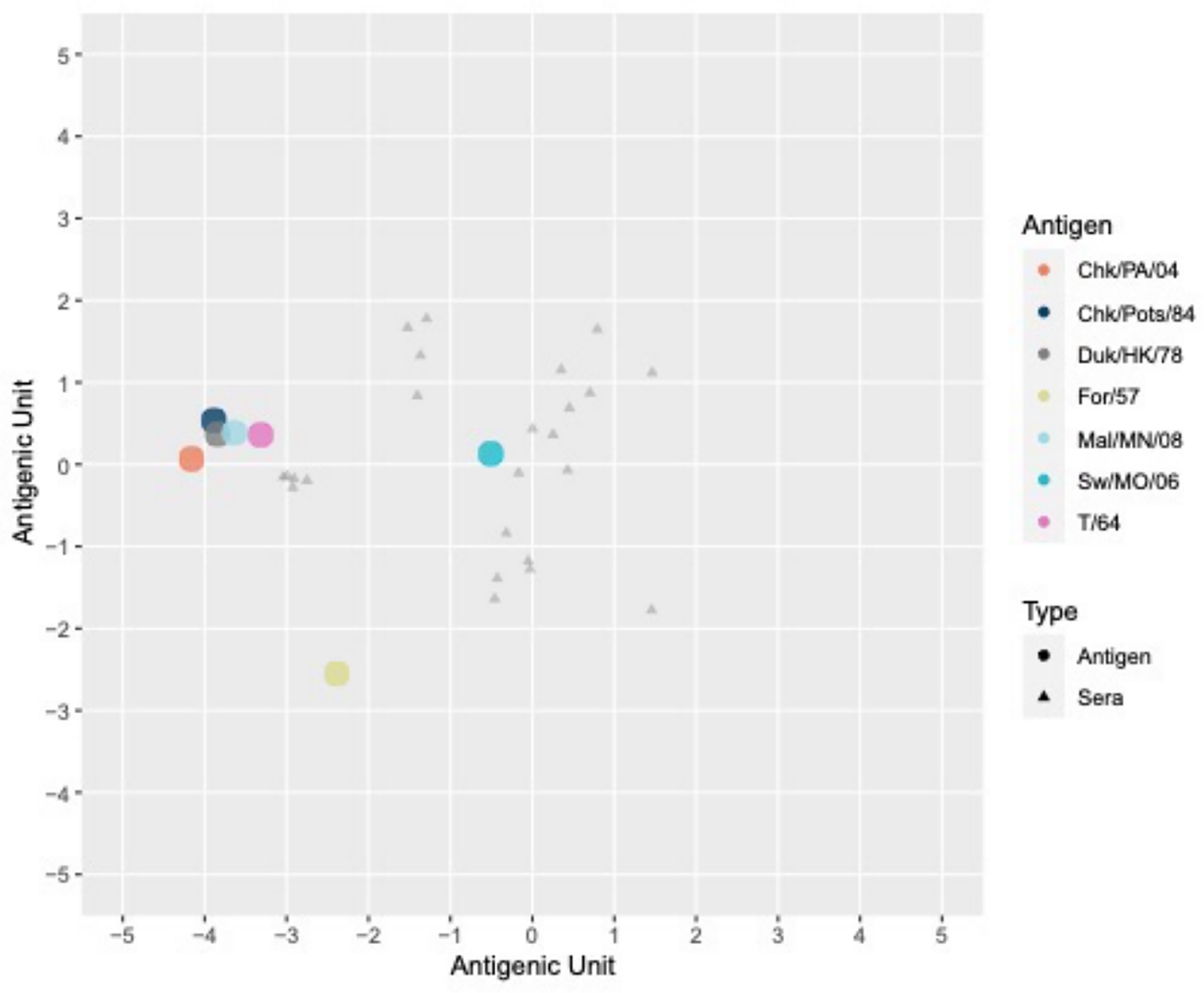

Supplement: Fig. S8 — Human neutralization antigenic cartography maps. [file msphere.00022-26-s0008.tif]
